# Comprehensive Cardiovascular and Renal Protection in Patients with Type 2 Diabetes

**DOI:** 10.3390/jcm12123925

**Published:** 2023-06-08

**Authors:** Almudena Castro Conde, Domingo Marzal Martín, Raquel Campuzano Ruiz, Maria Rosa Fernández Olmo, Carlos Morillas Ariño, Juan José Gómez Doblas, Jose Luis Gorriz Teruel, Pilar Mazón Ramos, Xavier García-Moll Marimon, Maria Jose Soler Romeo, David León Jiménez, Vicente Arrarte Esteban, Juan Carlos Obaya Rebollar, Carlos Escobar Cervantes, Juan J. Gorgojo Martínez

**Affiliations:** 1 Cardiology Department, University Hospital La Paz, 28046 Madrid, Spain; carlos.escobar@salud.madrid.org; 2 Cardiology Department, Hospital Quirónsalud San José, 28002 Madrid, Spain; domingo.marzal@gmail.com; 3 Cardiology Department, University Hospital Fundación Alcorcón, 28922 Madrid, Spain; raquel_campuzano@hotmail.com; 4 Cardiology Department, Complejo Hospitalario de Jaén, 23007 Jaén, Spain; mariarosafernandezolmo.urv@secardiologia.es; 5 Endocrinology Department, Hospital Doctor Peset, 46017 Valencia, Spain; cmorillas@telefonica.net; 6 Cardiology Department, University Hospital Virgen de la Victoria, 29010 Málaga, Spain; jjgomezdoblas@gmail.com; 7 Nephrology Department, University Hospital Clínico Valencia, 46010 València, Spain; jlgorriz@gmail.com; 8 Cardiology Department, Complejo Hospitalario Universitario Santiago de Compostela, 15706 A Coruña, Spain; pilarmazon@yahoo.es; 9 Cardiology Department, Hospital de la Santa Creu i Sant Pau, 08025 Barcelona, Spain; xgarcia-moll@santpau.cat; 10 Nephrology Department, Hospital Vall d’Hebron, 08193 Barcelona, Spain; m.soler@vhebron.net; 11 Internal Medicine Department, University Hospital Virgen del Rocío, 41013 Sevilla, Spain; davidleonj@yahoo.es; 12 Cardiology Department, University General Hospital, 03010 Alicante, Spain; viarrarte@hotmail.com; 13 Primary Care Center La Chopera, Alcobendas, 28100 Madrid, Spain; jcobaya69@gmail.com; 14 Department of Endocrinology and Nutrition, University Hospital Fundación Alcorcón, 28922 Madrid, Spain; juanjose.gorgojo@salud.madrid.org

**Keywords:** diabetes, cardiovascular, GLP-1 receptor agonists, glycated hemoglobin, renal, SGLT2 inhibitors

## Abstract

Type 2 diabetes (T2DM) is one of the main public health care problems worldwide. It is associated with a marked increased risk of developing atherosclerotic vascular disease, heart failure, chronic kidney disease and death. It is essential to act during the early phases of the disease, through the intensification of lifestyle changes and the prescription of those drugs that have been shown to reduce these complications, with the aim not only of achieving an adequate metabolic control, but also a comprehensive vascular risk control. In this consensus document, developed by the different specialists that treat these patients (endocrinologists, primary care physicians, internists, nephrologists and cardiologists), a more appropriate approach in the management of patients with T2DM or its complications is provided. A particular focus is given to the global control of cardiovascular risk factors, the inclusion of weight within the therapeutic objectives, the education of patients, the deprescription of those drugs without cardiovascular benefit, and the inclusion of GLP-1 receptor agonists and SGLT2 inhibitors as cardiovascular protective drugs, at the same level as statins, acetylsalicylic acid, or renin angiotensin system inhibitors.

## 1. Introduction

Diabetes mellitus (DM) is one of the main public health care problems, with nearly 540 million subjects with DM worldwide. However, this figure is expected to markedly increase in the coming years [1]. In Spain, the prevalence of diabetes in the adult population reaches 14%, of which half are undiagnosed [2].

Type 2 diabetes (T2DM) increases the risk of developing atherosclerotic vascular disease, heart failure (HF), chronic kidney disease (CKD) and of death [3]. This is especially important in patients with high or very high cardiovascular (CV) risk, which represent the majority of people with T2DM [4]. Therefore, it is essential to act in the early phases of the disease, through the intensification of lifestyle changes and the prescription of those drugs that have been shown to reduce these complications. As a result, the goal of treatment should be not only to achieve glycosylated hemoglobin (HbA1c) targets, but also to promote weight loss, improve control of other CV risk factors, and the use of those antihyperglycemic drugs that have demonstrated vascular-renal benefit [3,5,6]. In this way, the optimization of treatment through an overall and comprehensive approach to the patient with T2DM implies that the healthcare system should not delay the intervention until the patient has had a CV or renal event (a strategy formerly known as secondary prevention), but since cardiorenal risk is continuous, the double objective should be implemented early, as the benefit will be greater [7,8].

The aim objective of this document is to offer a more appropriate approach in the management of patients with T2DM or its complications, by health care professionals who attend this population, and thus improve their clinical evolution. This document updates previous recommendations [9,10] and has been endorsed by the Diabetes and Obesity working group of the Spanish Society of Cardiology.

## 2. Multifactorial Approach to Reduce the Vascular-Renal Risk

Patients with T2DM require intensive control of their risk factors, as they have a high risk of presenting serious CV complications throughout the evolution of the disease [3]. The efforts to achieve control targets should not be focused only on attaining HbA1c goals, but also on the control of other CV risk factors, including low-density lipoprotein cholesterol (LDLc), blood pressure, smoking, weight and lifestyle [3,5,6].

The STENO-2 study [11] showed that the multifactorial intensification of treatment of arterial hypertension, lipids, diet and physical exercise in patients with T2DM reduced the risk of presenting macro- and microvascular complications by 50%, but there were no significant differences in total mortality after a follow-up of 7.8 years. Surprisingly, these results did not have so much to do with glycemic control, since the target of HbA1C < 6.5% was only achieved in 15% of cases. In 2016, the results of the STENO-2 were published after a median follow-up of 21 years (13 years after the end of the study, with a duration of 7.8 years) [12]. Patients who had received the intensive multifactorial treatment had a 45% lower risk of death than those who had received the conventional treatment. The median survival of patients with intensive treatment was 7.9 years longer than that of patients with conventional treatment. Similarly, the median time to the first CV event was 8.1 years longer in intensively treated patients. In addition, the risk of progression to macroalbuminuria was 48% lower and the risk of the onset or progression of retinopathy was 33% lower in intensively treated patients. Remarkably, these results were obtained taking into account that during the last 13 years of the observational follow-up period, both groups received the same intensive treatment, which indicates the importance of the early achievement of the appropriate control of risk factors. 

Table 1 summarizes the targets for the different CV risk factors in subjects with T2DM [3,5,13].

### 2.1. Weight

Obesity is a chronic progressive condition with genetic, environmental, and behavioral determinants that result in excess associated adiposity. Obesity defined by the body mass index (BMI), and especially central obesity, is associated with a marked increase in morbidity and mortality [13]. In the Di@bet.es study, approximately 50% of patients with known DM had obesity defined as a BMI ≥ 30 kg/m^2^, which increased to 68% in the case of patients with abdominal obesity [2].

An evidence-based approach for the treatment of obesity incorporates lifestyle, medical, and surgical options, balances risks and benefits, and emphasizes medical outcomes that address the complications of obesity rather than cosmetic goals [13]. Weight loss should be considered in all patients with overweight or obesity with prediabetes or T2DM. Although a 5% weight loss with a reduction in waist circumference is associated with cardiometabolic benefits, a weight loss of at least 10% may reduce cardiovascular events. Furthermore, as demonstrated by the DIRECT study, weight loss >15% of body weight is associated with remission of T2DM [14]. Moreover, bariatric surgery has also been associated with reductions in CV morbidity and mortality and remission of T2DM [15,16]. Data from the PREDIMED study (Prevention with the Mediterranean Diet) [17] show that the Mediterranean diet reduces the development of T2DM by up to 40%, beyond weight loss (qualitative effect of the diet). The PREDIMED Plus study is currently comparing the hypocaloric Mediterranean diet vs. an isocaloric diet in patients with overweight/obesity and metabolic syndrome [18]. In summary, diets with adequate caloric restriction are necessary to lose weight, adapted to the pattern of the Mediterranean diet. The main characteristic of the diet should be the control of the amount of rapidly absorbed carbohydrates (sugar, sweets, juices, etc.) and the limitation of saturated and trans fats (red meats, sausages, pastries, etc.), but especially processed foods, as well as the increase in the intake of fruits, vegetables, legumes, extra virgin olive oil, nuts, whole grains, etc. [13,17].

### 2.2. Physical Activity

Physical activity delays the onset of T2DM in subjects with carbohydrate intolerance, improves glycemic control, and decreases the risk of developing CV complications in individuals with T2DM. Both aerobic and resistance exercise, and above all the combination of both types of exercise, improve insulin sensitivity and the control of different CV risk factors, including HbA1c, LDLc and blood pressure [19,20,21].

An individualized exercise prescription must be made, establishing the general characteristics of the aerobic exercise:

- Intensity level: it should be between 60% and 75% of what is called the cardiac reserve level. The simplest way to calculate exercise intensity is to use the Talking Test. In this case, it is about exercising hard enough that the person has difficulty having a conversation.
- Frequency and progression: the exercise must be carried out continuously. At least five days a week is recommended.
- Duration: A minimum of 30 min a day of moderate aerobic exercise is recommended, performed at least five days a week, or 90 min a week of high intensity exercise.

Additionally, resistance training not only enhances muscular strength and endurance, functional capacity, and quality of life, but also improves cardiovascular health. Stretching the major muscle or tendon groups, 2–3 days per week, is recommended. As a result, a combination of aerobic exercise and resistance exercise should also be recommended.

### 2.3. Smoking

It has been observed that one fifth of patients with DM are smokers [2]. Tobacco is, by itself, a risk factor that favors the appearance of T2DM [22]. Tobacco exponentially increases CV risk in patients with DM. The patient with DM that smokes doubles the risk of total mortality and greater CV events than the non-smoker patient with DM. However, this risk decreases when smoking is stopped. On the other hand, not only does active smoking increase CV risk; the same applies to passive smoking and certain tobacco products without combustion [23]. Furthermore, smoking also increases the risk of developing microvascular complications in patients with DM [24,25].

- Total cessation of tobacco use should be recommended.
- Particular attention should be given to supporting smokers in follow-up and offering them nicotine replacement therapy, cytisine and/or bupropion, as appropriate [26,27,28].
- Even if you gain weight, you should insist on cessation of tobacco, since the benefits of quitting smoking are greater [29].

### 2.4. Lipids 

The main objective in the treatment of dyslipidemia in patients with T2DM is the reduction in LDLc levels to the recommended targets, based on CV risk (Table 1). Furthermore, non-HDL cholesterol and apoB are included as secondary objectives. As an additional objective, in patients who have had a second CV event in the first two years (not necessarily of the same type), a LDLc target of <40 mg/dL may be considered [13].

To achieve these goals, high-intensity statins or the highest tolerated dose of statins are of choice. If LDLc is not controlled, ezetimibe could be added to the treatment. If the goals are still not achieved, especially in patients who have had an atherosclerotic CV event or have familial hypercholesterolemia, adding the subtilisin/kexin-type proprotein convertase inhibitors (PCSK9i) could be considered [13]. All these pharmacological groups have been shown to reduce the risk of CV events in the population with DM, by reducing LDLc levels [30,31]. More recently, bempedoic acid has been marketed, which has been shown to reduce LDLc and the risk of developing DM, as well as morbidity and mortality [32,33].

On the other hand, the REDUCE-IT trial [34] that included patients with CV disease or DM (57.8%) with another CV risk factor, LDLc between 40 and 100 mg/dL and triglycerides between 150 and 500 mg/dL, demonstrated a reduction of 25% with icosapent ethyl (4 g/day) in the risk of the primary endpoint (CV death, acute myocardial infarction, stroke, coronary revascularization, or unstable angina). Although icosapent ethyl reduces triglyceride levels by about 20%, the exact mechanism by which icosapent ethyl produces this clinical benefit is not well understood.

### 2.5. Blood Pressure

Arterial hypertension is common in patients with DM. In the Di@bet.es study, hypertension reached 80% of patients with known DM [2]. In addition, masked hypertension and the more harmful non-dipper pattern are also frequent in DM. Lowering blood pressure to recommended targets has been associated with a decrease in macro- and microvascular complications and mortality [35].

According to the European guidelines for arterial hypertension, antihypertensive drug treatment is recommended in hypertensive patients with DM when blood pressure is ≥140/90 mmHg, with a therapeutic goal of 120 -< 130 mmHg for systolic blood pressure (if tolerated), and 70 -< 80 mmHg for diastolic blood pressure [35].

The recommended first-line antihypertensive drugs are angiotensin-converting enzyme inhibitors (ACEi) or angiotensin II receptor blockers (ARBs), as they have shown additional benefits in this population, such as reduction in albuminuria and appearance or progression of diabetic nephropathy, more effectively than other antihypertensive agents [36]. Except in frail patients, or those with mild hypertension without target organ damage, the combination of a renin angiotensin system inhibitor with a calcium channel antagonist or a thiazide is recommended [35].

### 2.6. Glycated Hemoglobin

The current recommended target of HbA1c for patients with T2DM is <7%, or even lower (6.5%) in certain groups, as long as the drugs used for this purpose do not cause hypoglycemia and have proven CV safety [3]. There is a close relationship between achieving HbA1c targets and reducing microvascular complications, but not macrovascular complications or mortality [37]. In contrast, global control of CV risk factors has been associated with a reduction in CV complications [11].

As a result, HbA1c is an objective in the control of patients with T2DM, together with the control of the rest of the CV risk factors and the use of those drugs that have shown overall benefit [3]. On the other hand, new glucometric parameters obtained from continuous glucose monitoring are currently complementary to HbA1c and may even replace it in the near future as the main measure of glycemic control [38].

Several studies have identified severe hypoglycemia as a strong predictor of atherosclerotic CV disease, adverse clinical outcomes, and mortality in those with T2DM [39,40]. Hypoglycemia causes an increase in sympathetic system activity, leading to increased heart rate, cardiac stroke volume, and myocardial contractility as well as decreased peripheral resistance. In addition, it may induce an increased risk of arrhythmias and sudden death [40,41]. Therefore, it is crucial to attain HbA1c targets, but without causing severe hypoglycemia. 

### 2.7. Cognitive Impairment

Cognitive impairment should be assessed in all patients with T2DM. In fact, patients with T2DM have a higher risk of cognitive decline and an increased risk of dementia, mainly in untreated or poorly patients, and, conversely, patients with dementia have a higher risk of developing diabetes. In addition, in these patients the treatment should be simplified and safer antihyperglycemic drugs should be used to minimize the risk of hypoglycemia [42,43]. As a result, a comprehensive management is particularly relevant in the context of multimorbidity to reduce the risk of cognitive impairment in patients with T2DM, but also cognitive function should be taken into account when considering glycemic and CV risk factor targets and the most appropriate antihyperglycemic drugs [42,44].

## 3. Antihyperglycemic Drugs

Currently, antihyperglycemic drugs can be classified based on their ability to reduce the risk of developing CV and renal complications.

On the one hand, there are drugs without proven cardio-renal benefit, which would include metformin, sulfonylureas (SU), glinides, alpha-glucosidase inhibitors, dipeptidyl peptidase type 4 (DPP4i) inhibitors, pioglitazone and insulins [45].

On the other hand, there are drugs that have shown CV and renal benefit, which would include sodium-glucose cotransporter type 2 inhibitors (SGLT2i) and glucagon-like peptide 1 (GLP-1) receptor agonists [45]. The most relevant characteristics of each pharmacological group are summarized below.

### 3.1. Biguanides (Metformin)

Metformin is the only molecule currently marketed from the biguanide group and, together with pioglitazone, constitutes the therapeutic class of insulin-sensitizing drugs. Metformin reduces postprandial and basal plasma glucose and acts by three main mechanisms: (1) reduction in hepatic glucose production by inhibiting gluconeogenesis and glycogenolysis; (2) increase in insulin sensitivity at the muscle level, improving peripheral glucose uptake; (3) delay in intestinal absorption of glucose. In addition, by not stimulating insulin secretion, it does not cause hypoglycemia [46].

The UKPDS study showed in a small group of patients with recently diagnosed T2DM, overweight and low CV risk (*n* = 342) that compared to diet, metformin was able to significantly reduce the risk of myocardial infarction and mortality from all causes, after a median follow-up of 10.7 years. In addition, compared to standard treatments (SU or first-generation insulins), metformin reduced the risk of stroke and all-cause mortality [47]. However, a meta-analysis of clinical trials published in 2017 did not show a reduction in CV events or mortality with metformin [48].

Considering the extensive experience in clinical practice with metformin, the data from the UKPDS study, its safety, and its low cost, metformin had traditionally been considered the first-line drug in the therapeutic approach of patients with T2DM. However, as a result of the evidence from the latest clinical trials with SGLT2i and GLP-1 receptor agonists, in which the benefit of these drugs was independent of treatment with metformin, the latest recommendations have displaced it as the first-line drug, in favor of those antihyperglycemic drugs that have shown CV benefit [3,6].

Regarding HF, metformin has shown benefits only in observational studies and meta-analyses of observational studies, but not in randomized clinical trials [49]. The main limiting factors to the prescription of metformin are digestive tolerance and renal function. Lactic acidosis is a rare complication (5 cases/100,000 patients/year of treatment), mainly related to CKD. In this context, metformin can be used safely in patients with mild CKD and in some patients with moderate CKD. However, the drug would be contraindicated in patients with estimated glomerular filtration rates (eGFR) <30 mL/min/1.73 m^2^ and could be maintained with caution and monitoring of the renal function for eGFR between 45 and 30 mL/min/1.73 m^2^. It is not recommended to start treatment with metformin in patients with eGFR < 45 mL/min/1.73 m^2^ [3,46]. On the other hand, approximately 10–30% of patients treated with metformin show a deficit in vitamin B12 absorption which could cause blood or neurological disorders associated with low vitamin levels in patients with duration of treatment over 5–10 years. Consequently, it is recommended to determine vitamin B12 levels at least once a year [46].

### 3.2. Sulfonylureas

SU are secretagogue drugs, as they stimulate the production of insulin by the pancreas. Since the 1970s there has been some debate about their CV safety following results from the University Group Diabetes Program (UGDP), in which tolbutamide (first-generation SU) was associated with increased CV mortality [50]. However, in recent years, different meta-analyses and observational studies have been published with heterogeneous results. In some of these publications, SU do not seem to increase the number of coronary events, although they could increase the risk of strokes and have been associated with a significant increase in mortality [51,52].

However, in the ADVANCE study [49], an intensive glycemic control strategy, based on modified-release gliclazide together with other drugs to achieve a target HbA1c ≤ 6.5%, achieved a 10% relative reduction in the risk of a combined variable of macro/microvascular events, mainly due to a reduction in the risk of nephropathy, with a neutral effect on macrovascular complications. TOSCA-IT [53], a pragmatic study designed to compare the effect of pioglitazone versus SU in patients with T2DM not adequately controlled with metformin monotherapy, met the primary endpoint of death from all causes, non-fatal myocardial infarction, non-fatal stroke, or urgent coronary revascularization. Out of the 3028 enrolled patients (11% with prior CV disease), 1535 were assigned to pioglitazone and 1493 to SU: 2% to glibenclamide, 48% to glimepiride, and 50% to gliclazide. The study was stopped early after a median follow-up of 57 months because of futility. There were no differences in the primary endpoint between the groups (1.5% per year). The incidence of hypoglycemia was lower among the patients who received pioglitazone (10% vs. 34%; *p* < 0.001). In both groups there was a moderate increase in weight (less than 2 kg on average) and the same episodes of HF (1%), bladder cancer and fractures. Finally, CAROLINA [54] was a clinical trial in which linagliptin was compared to glimepiride in 6042 patients with T2DM and CV risk factors or established atherosclerotic CV disease, without finding significant differences in the risk of the primary variable composed of CV death, non-fatal myocardial infarction, or non-fatal stroke.

Therefore, to date SU have not been shown to be beneficial in reducing macrovascular complications in patients with T2DM and, in some cases, they could even be harmful, because of a higher risk of hypoglycemia.

### 3.3. Glinides

The glinides repaglinide and nateglinide exert their hypoglycemic effect by increasing insulin secretion and, together with the SU, they make up the group of so-called secretagogues. They regulate, like SU, the adenosine triphosphate (ATP)-dependent potassium channel of the pancreatic ß cell; however, their binding site is different and they are structurally distinct. They must be administered before meals because they control the early phase of insulin secretion. Unlike SU, which primarily reduce basal plasma glucose, glinides lower postprandial glycemia. Their efficacy in monotherapy is comparable to that of SU. Their main side effects are weight gain and hypoglycemia, both of which are minor compared to SU [55].

Studies about the CV safety of these drugs are scarce. In the NAVIGATOR study [56], conducted in 9306 subjects with carbohydrate intolerance and CV disease or risk factors, nateglinide did not reduce the incidence of DM or the risk of developing CV complications compared to a placebo, but increased the risk of hypoglycemia.

Therefore, at present there is not enough evidence available to confirm the CV safety of glinides. Their association with hypoglycemia and their CV profile, similar to that of SU in a single study, discourage their use.

### 3.4. α-Glucosidase Inhibitors

The α-glucosidase inhibitors, acarbose and miglitol, inhibit the cleavage of large carbohydrate molecules in the gastrointestinal tract, delay their absorption, and reduce postprandial blood glucose. In addition, it has been described that their administration increases GLP-1 levels and can alter the intestinal microbiota. The mean decrease in HbA1c with these molecules is 0.5–0.7%. In addition, these drugs contribute to lowering postprandial triglycerides, but have no effect on fasting triglycerides or on HDL or LDL cholesterol levels. Similarly, no significant effects on blood pressure or weight have been described. The main side effects are meteorism and diarrhea [57].

The STOP-NIDDM [58] study evaluated the CV risk of patients with impaired glucose tolerance, as well as the risk of developing T2DM. For this purpose, 1429 patients were randomized to receive acarbose or a placebo in a multicenter study. The mean follow-up was 3.3 years. In the group of patients who received acarbose, the relative risk of developing diabetes was reduced by 25%, hypertension by 34%, as well as CV events by 49% compared to the placebo. However, the number of events was small and lacked statistical power [59]. Likewise, in the ACE study [60] that included Chinese subjects ≥50 years, with established coronary artery disease and prediabetes, acarbose did not reduce the risk of developing CV complications compared with a placebo.

Therefore, acarbose has been shown to be safe from a CV point of view in these patients, but has not been definitely proven to have a CV benefit.

### 3.5. Thiazolidinediones or Glitazones

Glitazones activate nuclear peroxisome proliferator-activated receptor ƴ receptors and exert their action through three known mechanisms: (1) improvement in insulin resistance with little or no tendency to produce hypoglycemia; (2) increase in glucose uptake by skeletal muscle through the regulation of adipocyte cytokines responsible for insulin sensitization; and (3) decrease in hepatic glucose production. However, they also cause hydrosaline retention and peripheral edema, increasing weight and the risk of HF [61]. Consequently, this pharmacological group is contraindicated in patients with HF or the risk of developing HF [61]. In addition, rosiglitazone was withdrawn from the European market by the EMA due to an increased risk of myocardial infarction. In the United States it is still on the market, although as a last therapeutic alternative. Currently, in Europe only pioglitazone is available [62].

Regarding pioglitazone, in the PROACTIVE [63] study pioglitazone was compared with a placebo in 5238 patients with T2DM and established CV disease. Although there were no significant differences in the primary endpoint (death from all causes, non-fatal myocardial infarction, stroke, acute coronary syndrome, endovascular or surgical intervention in the coronary or leg arteries, and amputation above the ankle), pioglitazone significantly reduced the secondary endpoint of all-cause mortality, myocardial infarction, or nonfatal stroke by 16% after a mean follow-up of 34.5 months (NNT = 49). However, pioglitazone increased the risk of hospitalization for HF by 40% (NNH = 62). The IRIS [64] study evaluated the safety and efficacy of pioglitazone in 3876 patients with insulin resistance (without T2DM) and strokes or transient ischemic attacks in the last six months. Pioglitazone decreased the primary composite endpoint of fatal and nonfatal myocardial infarction or stroke by 24%, after a median follow-up of 4.8 years (NNT = 36). However, treatment with pioglitazone was associated with a significantly higher frequency in the number of edema (NNH = 9.3) and bone fractures (NNH = 53).

Even though some societies recommend pioglitazone for its beneficial CV effects (MACE3 in PROACTIVE or stroke in IRIS), it also increases the risk of HF. Therefore, as a general rule, pioglitazone should not be recommended in patients with T2DM and a high risk of HF. It is unknown if lower doses of pioglitazone (15 or 30 mg daily) than that used in the PROACTIVE trial (45 mg daily) could change the benefit/risk balance of this drug regarding HF.

### 3.6. Inhibitors of the Enzyme Dipeptidyl Peptidase Type 4

The mechanism of action of DPP4i consists of avoiding the inactivation of GLP-1 in order to potentiate and prolong the effects of the endogenous release of this hormone, substantially improving fasting and postprandial glycemic control, without producing hypoglycemia [65].

All CV outcomes studies with DPP4i have failed to show any CV benefit. TECOS [66] is the study with the longest follow-up. This study showed that with a similar glycemic control, there were no differences in any of the defined CV endpoints (main CV endpoint composed of CV death, new myocardial infarction, stroke, or admission for unstable angina) or admissions for HF.

The FDA published a communication warning about the association of HF with the use of saxagliptin and alogliptin [67], despite the fact that the EXAMINE [68] study did not find a significant difference in the increase in HF in the alogliptin group, but it was observed in the SAVOR-TIMI 53 [69] study with saxagliptin.

The CARMELINA [70] study did not show significant differences in the composite outcome MACE-3 between linagliptin and a placebo. In addition, rates of hospitalization for HF and severe renal events (renal death, end-stage renal disease, or eGFR ≥ 40% lower than baseline) were also similar between both groups. Similar results were found in the CAROLINA [54] study comparing linagliptin vs. the SU glimepiride.

In summary, DPP4i is a drug class that has been shown to be neutral from a CV point of view, and some of them could even increase the risk of HF.

### 3.7. Sodium-Glucose Cotransporter Type 2 Inhibitors

SGLT2i selectively, potently, and reversibly inhibit SGLT2. SGLT2 inhibition results in reduced glucose reabsorption in the renal proximal convoluted tubule, concomitantly decreasing sodium reabsorption. All of this promotes increased urinary glucose excretion, natriuresis, osmotic diuresis, and a reduction in intraglomerular pressure, which ultimately leads to a decrease in volume overload, blood pressure, body weight, preload and afterload, inducing reverse cardiac remodeling and preservation of the renal function. Increased urinary glucose excretion improves fasting and postprandial plasma glucose levels. However, the amount of glucose eliminated by the kidney through this mechanism depends on the concentration of glucose in the blood and glomerular filtration. Thus, in people with normal blood glucose, the glucose-lowering effect is low, and similarly, as the glomerular filtration decreases, the effect on glycosuria is also lower. Additionally, an improved homeostasis for beta cell function has also been observed [71,72].

Until September 2015, the date on which the EMPA-REG OUTCOME [73] study was published, no antihyperglycemic drug had shown a reduction in CV risk. Consequently, this study changed the paradigm in the treatment of T2DM. In this study, 7028 patients with long-standing T2DM and established CV disease were randomized to receive empagliflozin or a placebo. The primary endpoint was the composite endpoint of CV mortality, nonfatal myocardial infarction, or nonfatal stroke. The study was event-driven and lasted 3.1 years. Empagliflozin significantly reduced the primary endpoint by 14%, with no significant differences in the rates of myocardial infarction or stroke. Furthermore, in the empagliflozin group, a relative risk reduction of 35% for hospitalization for HF was found, as well as a significant reduction in CV mortality of 38% and total mortality of 32% compared to the placebo. The renal substudy of EMPA-REG [74] also demonstrated a nephroprotective effect of empagliflozin, with a 39% risk reduction in the progression of renal failure. Empagliflozin also reduced the occurrence of albuminuria and the need for dialysis.

In 2017, the CANVAS [75] program with canagliflozin was published, which integrated data from two clinical trial studies and included 10,142 individuals with T2DM and high CV risk. The primary endpoint was composed of CV death, non-fatal myocardial infarction, and non-fatal stroke. Treatment with canagliflozin was associated with a reduction in the primary study endpoint of 14% versus the placebo and the risk of hospitalization for HF by 33%. Likewise, the renal results showed a benefit of canagliflozin in the progression of albuminuria and the renal combined objective composed of a 40% sustained reduction in the eGFR, the need for renal transplantation and death from renal causes.

The DECLARE TIMI 58 [76] study evaluated the effect of dapagliflozin versus a placebo in 17,160 patients with T2DM with CV risk factors or CV disease (40.6% of the total population). This study differs from the previous ones in that it is the first with two co-primary objectives: a composite objective of reduction in CV death, non-fatal myocardial infarction and non-fatal stroke, and on the other hand, a reduction in CV death or hospitalization for HF. Secondary efficacy endpoints included a combined renal endpoint (40% reduction in eGFR, end-stage renal disease, renal or CV death) and all-cause mortality. The mean follow-up was 4.2 years. Dapagliflozin met the non-inferiority endpoint compared to the placebo in reducing the MACE endpoint, but did not show superiority in any of the primary or secondary prevention subgroups. In contrast, the co-primary endpoint of CV death or hospitalization for HF was significantly reduced by dapagliflozin (4.9% vs. 5.8%; HR: 0.83; 95% CI: 0.73–0.95; *p* = 0.005), mainly due to a reduction in HF hospitalizations (HR: 0.73; 95% CI: 0.61–0.88). There were no significant differences in CV mortality (HR: 0.98; 95% CI: 0.82–1.17). Furthermore, there was a reduction in renal events in the group treated with dapagliflozin compared to the group treated with a placebo (4.3% vs. 5.6%; HR: 0.76; 95% CI: 0.67–0.87). The benefit of dapagliflozin in reducing the combined endpoint of CV death or HF admission was highly consistent across the different subgroups and independent of the history of established CV disease.

Not all SGLT2i have shown benefits in terms of reducing CV events in patients with T2DM. Thus, the VERTIS-CV [77] study analyzed the CV safety of ertugliflozin versus a placebo in patients with T2DM and established CV disease. Although ertugliflozin met the primary endpoint of non-inferiority for MACE, it did not show superiority for MACE. There were no significant differences between the two doses of ertugliflozin, 5 and 15 mg. The combined secondary endpoint of CV death or HF hospitalization was also not significant. However, there was a decrease in the risk of hospitalization for HF, although this result was not considered statistically significant due to the hierarchical analysis of the study. Additionally, no significant differences between groups were observed in the combined renal endpoint (composite of renal death, dialysis/transplantation, or doubling of serum creatinine). Other SGLT2i marketed outside USA or the European Union have not published CV outcomes trials yet.

Although previous studies conducted in the populations with T2DM diabetic population suggested a renal benefit in secondary endpoints, the CREDENCE [78], DAPA-CKD [79] and EMPA-KIDNEY [80] studies have specifically demonstrated significant benefits of SGLT2i in patients with CKD.

The CREDENCE [78] study included patients with advanced CKD, T2D, and overt albuminuria, with a high proportion of patients (60%) with reduced eGFR, who were randomized to treatment with canagliflozin or a placebo. The patients had eGFR between 30 and 90 mL/min/1.73 m^2^ and albuminuria, mostly greater than 300 mg/g. This study was the first to use a renal primary endpoint, a composite endpoint of end-stage renal disease (dialysis for at least 30 days, kidney transplant or eGFR < 15 mL/min/1.73 m^2^ sustained for at least 30 days according to measurements analyzed in the central laboratory), doubling of baseline serum creatinine sustained for at least 30 days or death from renal or CV causes. The study was stopped early after a pre-specified interim analysis due to significant benefit in the active treatment group, with a 30% reduction in the primary endpoint in favor of canagliflozin (HR: 0.70; 95% CI 0.59–0.82; *p* = 0.00001). Results for CV secondary endpoints were also favorable for canagliflozin treatment. They are shown below in order of hierarchical analysis after having achieved the primary objective: the composite endpoint of CV death or HF admission was reduced by 31% (HR 0.69; 95% CI 0.57–0.83; *p* < 0.001); the composite of CV death, myocardial infarction or non-fatal stroke showed a 20% risk reduction (HR 0.61; 95% CI 0.47–0.80; *p* < 0.001); HF hospitalizations were also reduced by 39% (HR 0.61; 95% CI 0.47–0.80; *p* < 0.001); the composite of end-stage renal disease, doubling of baseline serum creatinine or renal death was reduced by 34% (HR 0.66; 95% CI 0.53–0.81; *p* < 0.001); and CV mortality was reduced by 22% (HR 0.78; 95% CI 0.61–1.00; *p* = 0.05). In addition, there was also a trend towards a lower risk of all-cause mortality (HR 0.83; 95% CI 0.68–1.02). The results were consistent in the different subgroups analyzed. On the other hand, no significant differences were observed in terms of amputation or fracture rates. This was an important point, as the patients included in this study were at higher risk than those included in the CANVAS study [75] (in which a significant increase in amputations and fractures in patients treated with canagliflozin was reported).

In the DAPA-CKD study [79], among patients with an eGFR of 25 to 75 mL/min/1.73 m^2^ and an urinary albumin-to-creatinine ratio of 200 to 5000 mg/g, with or without T2DM, dapagliflozin reduced CKD worsening, defined as the composite of a sustained decline in the eGFR of at least 50%, end-stage kidney disease, or death from renal or CV causes by 39% compared to a placebo when added to standard treatment, with an NNT of 19 to prevent 1 event of primary outcome after a median follow-up of 2.4 years (HR 0.61; 95% CI: 0.51–0.72; *p* < 0.001). There was also benefit in secondary endpoints: a 31% risk reduction in all-cause mortality (HR 0.69; 95% CI: 0.53–0.88; *p* = 0.0035), a 29% reduction in the risk of hospitalization for HF or CV death (HR 0.71; 95% CI: 0.55–0.92; *p* = 0.0089) and a 44% reduction in the risk of worsening renal function or renal death (HR 0.56, 95% CI: 0.45–0.68, *p* < 0.0001). The benefit was similar between patients with and without T2DM.

The EMPA KIDNEY study [80] included 6609 patients with CKD, defined as an eGFR of 20 -< 45 mL/min/1.73 m^2^ or 45 -< 90 mL/min/1.73 m^2^ with a urinary albumin-to-creatinine ratio of at least 200 mg/g. Patients received empagliflozin (10 mg once daily) or a placebo. The primary outcome was a composite of progression of kidney disease (defined as end-stage renal disease, a sustained decrease in eGFR to <10 mL/min/1.73 m^2^, a sustained decrease in eGFR of ≥40% from baseline, or death from renal causes) or death from cardiovascular causes. After a median follow-up of 2.0 years (the study was completed earlier than estimated), empagliflozin reduced the primary endpoint compared to the placebo (13.1% vs. 16.9%; HR: 0.72; 95% CI, 0.64 to 0.82; *p* < 0.001), regardless of the diabetes status and across different ranges of renal function. Additionally, rates of hospitalization from any cause were lower with empagliflozin (HR: 0.86; 95% CI, 0.78 to 0.95; *p* = 0.003), but not the composite outcome of HF hospitalization or death from cardiovascular causes or death from any cause. Remarkably, this study showed that empagliflozin was able to slow the progression of glomerular filtration in patients with or without T2DM, but also, for the first time, with or without albuminuria.

The SCORED study [81] was a clinical trial that included 10,584 patients with T2DM and CKD (eGFR of 25–60 mL/min/1.73 m^2^, with or without albuminuria) and risk of CV disease. Patients were randomized to sotagliflozin (not marketed in the European Union), a dual SGLT1/SGLT2i, or a placebo. The primary endpoint was changed during the trial to the combination of total number of CV deaths, HF hospitalizations, and HF emergency visits. The study was terminated early due to loss of funding. After a median follow-up of 16 months, the event rate for the primary endpoint was 5.6 events/100 patient-years in the sotagliflozin group and 7.5 events/100 patient-years in the placebo group (HR 0.74; 95% CI: 0.63–0.88, *p* < 0.001). The CV death rate per 100 patient-years was 2.2 with sotagliflozin and 2.4 with a placebo (HR 0.90; 95% CI 0.73–1.12; *p* = 0.35). For the original co-primary endpoint of first-occurrence CV death, non-fatal myocardial infarction, or non-fatal stroke, the HR was 0.84 (95% CI 0.72–0.99); for the original co-primary endpoint of first-occurrence CV death or HF hospitalization, the HR was 0.77 (95% CI: 0.66–0.91). Diarrhea, genital fungal infections, volume depletion, and diabetic ketoacidosis were more common with sotagliflozin. Thus, in patients with T2DM and CKD, with or without albuminuria, sotagliflozin resulted in a lower risk of the combined endpoint of CV deaths, HF hospitalizations, and HF emergency visits than the placebo, but was associated with a greater number of adverse events.

Another group of patients in which SGLT2i has proven to be especially beneficial is in subjects with HF, regardless of the presence of T2DM. It was initially demonstrated in HF subjects with reduced ejection fraction, and more recently also in HF patients with preserved ejection fraction.

The DAPA-HF study [82] included 4744 patients with HF and ejection fraction <40% (New York Heart Association [NYHA] II-IV) and evaluated the efficacy of dapagliflozin 10 mg versus a placebo in reducing the composite endpoint of CV death, hospitalization for HF or the need for assistance with intravenous treatment for HF. The mean follow-up was 18.2 months. The primary composite endpoint occurred in 16.3% of the dapagliflozin group and 21.2% of the placebo group (HR 0.74; 95% CI 0.60–0.85; *p* < 0.001). The first hospitalization for HF occurred in 237 patients with dapagliflozin (10.0%) and 326 with the placebo (13.7%) (HR 0.70; 95% CI: 0.59–0.83). Likewise, CV death was significantly lower with dapagliflozin (9.6%) than with the placebo (11.5%) (HR 0.82; 95% CI: 0.69–0.98). Regarding death from any cause, it was also lower in the dapagliflozin group (11.6%) than in the placebo group (13.9%) (HR 0.83; 95% CI: 0.71–0.97). The results were homogeneous in all subgroups and the adverse events were similar in both groups. A relevant fact is that this benefit was observed both in patients with and without T2DM.

The EMPEROR-Reduced [83] demonstrated the superiority of empagliflozin versus a placebo in HF patients with an ejection fraction <40%, and NYHA functional class II to IV, for the primary endpoint of CV death or HF hospitalization (HR 0.75, 95% CI: 0.65–0.86, *p* < 0.001), regardless of the presence of T2DM. The hospitalization rate for HF was lower in the empagliflozin group than in the placebo group (HR 0.70; 95% CI: 0.58–0.85; *p* < 0.001), but there were no differences in CV mortality between both groups. However, the annual rate of decline in eGFR was slower in the empagliflozin group than in the placebo group (−0.55 vs. −2.28 mL/min/1.73 m^2^; *p* < 0.001).

The SOLOIST-WHF study [84] evaluated sotagliflozin versus a placebo in patients with a history of T2DM and one hospitalization for HF, regardless of ejection fraction. The primary endpoint of the study was CV mortality and HF hospitalizations. Unfortunately, recruitment to the study was suspended in March 2020 due to lack of funding from the sponsor, so it was necessary to change this objective to the number of deaths from CV causes, hospitalizations and emergency department visits for HF, in order to increase the power of the study. The secondary objective included these same individually assessed outcomes and the occurrence of myocardial infarction, stroke, changes in quality of life assessed by the Kansas questionnaire, and changes in eGFR. Patients included in the study were randomly assigned, during hospitalization or within 3 days after discharge, to treatment with sotagliflozin 200 mg with a post-dose titration to 400 mg or a placebo. The mean follow-up was 9.2 months and 97.1% completed the follow-up. Of the 1222 randomized patients, 79.1% had an ejection fraction less than 50%. This study found a 33% relative risk reduction in the primary endpoint (HR 0.67; 95% CI 0.52–0.85; *p* < 0.001) and a 36% risk reduction in HF hospitalizations (HR 0.64, 95% CI 0.49–0.83, *p* < 0.001) with sotagliflozin. However, no significant reductions in CV or other-cause mortality were observed. In the subgroup analysis, the effect was found to be consistent in patients with ejection fraction greater than and less than 50%, as well as in patients who started therapy in hospital or on discharge.

The EMPULSE study [85] included 530 patients (mean age 68 years) hospitalized for acute HF (de novo or decompensated chronic HF), regardless of ejection fraction. Patients were randomized to receive empagliflozin or a placebo. After 90 days of follow-up, compared with the placebo, empagliflozin reduced the primary composite endpoint of CV mortality, HF hospitalizations, or improvement in quality of life (defined as an improvement of at least 5 points on the Kansas City Questionnaire) by 36%, as well as the individual components.

Therefore, the results of these studies support the use of SGLT2i in a new scenario: patients hospitalized for HF or after early discharge.

Regarding HF patients with preserved ejection fraction, the EMPEROR-Preserved study [86] evaluated the efficacy and safety of the administration of empagliflozin 10 mg versus a placebo in HF patients with preserved ejection fraction defined as ejection fraction greater than 40%, with proven elevation of natriuretic peptides (NT-proBNP greater than 300 pg/mL in sinus rhythm or greater than 900 pg/mL in atrial fibrillation) and NYHA II-IV functional class. A total of 5988 patients were enrolled to receive empagliflozin (10 mg once daily) or a placebo in addition to their usual therapy. The primary endpoint was a combination of cardiovascular death or HF hospitalization. After a median follow-up of 26.2 months, the primary endpoint occurred in 415 of 2997 patients (13.8%) in the empagliflozin group and in 511 of 2991 patients (17.1%) in the placebo group. This effect was mainly related to a lower risk of hospitalization for HF in the empagliflozin group. The NNT was 31 patients with empagliflozin, with a relative risk reduction of 21%. For secondary endpoints, the total number of HF hospitalizations was lower in the empagliflozin group than in the placebo group, with a relative risk reduction of 29%. eGFR decline was lower in the group assigned to empagliflozin (−1.25 vs. −2.62 mL/min/year). The effects of empagliflozin were consistent in patients with or without T2DM.

The DELIVER study [87] has recently been published. In this study, 6263 patients with HF with an ejection fraction of more than 40% were included. Patients received dapagliflozin 10 mg/day or a placebo, added to standard therapy. The primary endpoint was the combination of cardiovascular death or worsening HF, defined as hospitalization for HF or a visit to the emergency department for HF. After a median follow-up of 2.3 years, the primary endpoint occurred in 16.4% of dapagliflozin-treated patients and 19.5% of the placebo group (HR 0.82; 95% CI 0.73–0.92; *p* < 0.001). HF worsening occurred in 11.8% of patients treated with dapagliflozin and 14.5% with the placebo (HR 0.79; 95% CI 0.69–0.91); CV mortality in 7.4% and 8.3%, respectively (HR 0.88; 95% CI 0.74–1.05). The symptoms also improved with dapagliflozin. The results were similar in the groups with ejection fraction ≥60% and <60%, and were also independent of the presence of T2DM. The incidence of adverse effects was similar in both treatment groups.

In summary, SGLT2i have changed the paradigm of treatment, with CV benefits not only in the population with DM, but also in subjects with CKD and HF, regardless of the ejection fraction and the presence of T2DM.

### 3.8. Glucagon-Like Peptide 1 Receptor Agonists

GLP-1 receptor agonists, together with iDPP-4, make up the group of incretins. They activate the GLP-1 receptor, thereby stimulating endogenous glucose-dependent insulin secretion. In addition, they inhibit glucagon secretion, slow gastric emptying, increase satiety, decrease appetite and promote weight loss. They have extraglycemic effects, as they are associated with a reduction in blood pressure and an improvement in ventricular and endothelial function, improve the response to cardiac ischemia, and decrease hepatic lipogenesis, thereby improving hepatic steatosis. Due to their mechanism of action, dependent on the presence of glucose, they are associated with a low risk of hypoglycemia. The most frequent side effects are gastrointestinal, mainly nausea, vomiting and diarrhea. Currently, we have GLP-1 receptor agonists for daily subcutaneous administration (liraglutide, exenatide, lixisenatide) and weekly (dulaglutide, semaglutide) and more recently oral administration (semaglutide). In addition, the FDA and the EMA have authorized the marketing of tirzepatide (a GLP-1/GIP dual agonist for weekly administration), which has the greatest efficacy in glycemic control and weight loss of all current antihyperglycemic drugs [88]. In Spain it is not yet available.

From the perspective of CV benefit, the results of the pivotal studies with lixisenatide, exenatide, liraglutide, semaglutide, albiglutide, dulaglutide, and efpeglenatide have now been published. It should be noted that lixisenatide and exenatide once-weekly showed a neutral result in terms of CV benefit, although the rest of the drugs did show this benefit [89,90]. Albiglutide and exenatide LAR have been withdrawn from the market and efpeglenatide has not been marketed yet.

The LEADER study [91] evaluated the effect of liraglutide in patients with T2DM at high CV risk. A total of 9340 patients were evaluated over 3.5–5 years and were randomly assigned to receive liraglutide 1.8 mg by subcutaneous injection once daily (or the maximum tolerated dose) or a placebo, in addition to standard treatment. After a median follow-up of 3.8 years, liraglutide reduced the risk of the primary endpoint, which consisted of CV death, non-fatal myocardial infarction (including silent), and non-fatal stroke, by 13% (NNT = 55). The reduction in CV mortality was 22% (NNT = 79) and the decrease in all-cause mortality was 15% (NNT = 71). The rates of the other components of the primary endpoint (myocardial infarction, stroke, admission for chronic HF) were not statistically different from the placebo group. Therefore, LEADER was the first study with GLP-1 receptor agonists that demonstrated a reduction in mortality. Liraglutide also reduced HbA1c, body weight, and hypoglycemia, and the adverse effects were similar to those observed in previous studies, the most frequent being gastrointestinal.

These results were supported by the publication of SUSTAIN-6 [92] with semaglutide, which has a long half-life and only needs to be injected subcutaneously once a week. The study included 3297 patients and obtained a favorable result to active treatment in the primary endpoint composed of CV death, non-fatal myocardial infarction and non-fatal stroke of 26% (NNT = 43), of stroke of 39% (NNT = 97) and coronary or peripheral revascularization of 35%. Rates of new or worsening nephropathy were lower in the semaglutide group, but rates of retinopathy complications (vitreous hemorrhage, blindness, or conditions requiring treatment with an intravitreal agent or photocoagulation) were significantly higher (HR, 1.76, 95% CI 1.11–2.78, *p* = 0.02). Semaglutide was also shown to be a potent antihyperglycemic agent, with large and sustained reductions in HbA1c concentrations compared to a placebo, and similar rates of hypoglycemia, although glucose lowering was not the primary endpoint of the study. There was no difference in CV deaths between the study arms: semaglutide 0.5 mg once weekly, semaglutide 1.0 mg once weekly, and the placebo. The increased incidence of retinopathy events does not seem to be attributable to semaglutide, but rather to the rapid drop in HbA1c in patients with pre-existing retinopathy and poor glycemic control, a phenomenon that has been previously observed in patients with type 1 DM, pregnant women, and patients undergoing bariatric surgery.

Semaglutide is the only GLP-1 receptor agonist that has an oral formulation. The PIONEER-6 study [93] included 3183 patients with T2DM and high CV risk, defined as age ≥50 years with established CV disease or CKD, or age ≥60 years with at least one CV risk factor. The primary endpoint of the study was the combination of CV death, nonfatal myocardial infarction, or nonfatal stroke. After a median follow-up of 15.9 months, there was a trend for a lower risk of the primary study endpoint in favor of oral semaglutide (target dose 14 mg) vs. the placebo (3.8% vs. 4.8%; HR 0.79, 95% CI 0.57–1.11, *p* < 0.001 for non-inferiority). Importantly, although this study was not designed to demonstrate superiority in reducing major CV events, a reduction in CV mortality was observed (HR 0.49; 95% CI 0.27–0.92; *p* = 0.03) and all-cause mortality (HR 0.51; 95% CI 0.31–0.84; *p* = 0.008) with oral semaglutide. HbA1c, weight, and blood pressure reductions were in line with studies performed with subcutaneous semaglutide. Although permanent discontinuation due to serious adverse events was lower with oral semaglutide (2.6% vs. 3.0%), gastrointestinal adverse events leading to discontinuation were more frequent with oral semaglutide (6.8% vs. 1.6%), the majority of them being non-serious adverse events. Rates of severe hypoglycemia were uncommon, occurring in patients additionally taking insulin or sulfonylureas. The SOUL study (NCT03914326), a superiority study with oral semaglutide that includes more than 9000 patients with T2DM and established CV disease or CKD, is still ongoing. In addition, there are currently ongoing two randomized clinical trials, with the aim of determining the effect of semaglutide on the progression of CKD in patients with T2DM; the FLOW (NCT03819153) and the REMODEL (NCT04865770) clinical trials. This last study includes renal resonance in all patients and in some of them renal biopsy at baseline and at the end of treatment.

EXSCEL [94] was a pragmatic, randomized, placebo-controlled, double-blind clinical trial in which participants were randomized to receive exenatide 2 mg once weekly or a placebo. This study was designed to characterize the effects of weekly exenatide, in CV outcomes in 14,752 patients with T2DM, when added to their usual treatment. Patients were stratified by the history of CV disease (73.1% in secondary prevention). The primary endpoint was time to CV death, non-fatal myocardial infarction, or non-fatal stroke. Fewer episodes (without significant differences) were observed in the weekly exenatide group than in the placebo group: 11.4 vs. 12.2%, HR: 0.91; 95% CI: 0.83–1.00; *p* = 0.06. However, in the weekly exenatide group there was a 14% relative risk reduction in all-cause mortality (6.9 vs. 7.9%, respectively) (HR 0.86; 95% CI: 0.77–0.97, *p* = 0.016). Although there was a significant decrease in all-cause mortality in patients treated with weekly exenatide, this cannot be considered as such due to the prespecified hierarchical analysis of the study. In fact, once-weekly exenatide has been recently withdrawn from the market.

In 2018, the HARMONY OUTCOMES study [95] was published, in which 9463 patients with T2DM and CV disease, 70% of those with coronary disease, were assigned to receive 30–50 mg weekly of albiglutide or a placebo. After a follow-up of 1.6 years, the primary outcome defined as CV death, non-fatal myocardial infarction, or non-fatal stroke was significantly reduced with albiglutide (HR: 0.78; 95% CI: 0. 68–0.90) (non-inferiority, *p* < 0.0001; superiority, *p* = 0.0006). This reduction was mainly due to a decrease in the rate of fatal and non-fatal myocardial infarction (HR: 0.75; 95% CI: 0.61–0.90). There was no reduction in CV mortality. Secondary endpoints of HF hospitalization and all-cause mortality were not significantly different between groups. It should be noted that albiglutide has been withdrawn from the market by decision of the company itself, citing commercial reasons before the publication of this study.

The REWIND study [96] included 9901 patients with T2DM, who were assigned to receive the maximum dose of dulaglutide (1.5 mg weekly) or a placebo. The primary endpoint was a composite of CV events (CV death, nonfatal myocardial infarction, and nonfatal stroke) and the secondary endpoint, a composite of microvascular abnormalities (diabetic retinopathy or CKD). The initial median duration of diabetes was 10.5 years, the median baseline HbA1c was 7.2%, and 31.5% of the population had established CV disease. Participants were followed for almost 5.4 years. During this period, the primary outcome was observed in 12.0% of subjects assigned to the dulaglutide group, compared to 13.4% of those assigned to the placebo (HR: 0.88; 95% CI: 0.79–0.99; *p* = 0.026). Separating each component of the primary endpoint, it is noteworthy that the difference between the groups was related to a lower rate of non-fatal strokes: 2.7% with dulaglutide and 3.5% with the placebo (HR: 0.76; 95% CI: 0.61–0.95, *p* = 0.017). 

The AMPLITUDE-O study [97] included 4076 patients with T2DM and either a history of CV or CKD, defined as eGFR of 25–59.9 mL/min/1.73 m^2^ and at least one other CV risk factor. Patients were randomized to efpeglenatide 4 mg or 6 mg sc weekly, or a placebo. The primary endpoint was the occurrence of major CV events, composed of myocardial infarction, non-fatal stroke, or all-cause or CV death. After a follow-up of 1.8 years, efpeglenatide reduced the risk of MACE by 27% (7% vs. 9.2%; HR 0.73; 95% CI 0.58–0.92). Renal events, described as impaired renal function or macroalbuminuria, were also lower in the efpeglenatide group (13% vs. 18%; HR 0.68; 95% CI 0.57–0.79; *p* < 0.001). Diarrhea, constipation, nausea, and vomiting were more common in patients assigned to efpeglenatide. Efpeglenatide has not been marketed yet. 

The SURPASS-CVOT (NCT04255433) is a clinical trial currently ongoing that is comparing the efficacy of tirzepatide vs. dulaglutide on major cardiovascular events in patients with T2DM.

Table 2 summarizes the clinical trials that have shown CV benefit in patients with T2DM.

### 3.9. Insulin

Currently, treatment with insulin in T2DM is reserved for those patients with severe symptoms, a high likelihood of T1DM, low pancreatic beta-cell mass, or in whom control objectives have not been achieved with other treatments. Several types of insulin are available according to their duration of action, and their combination can, at least potentially, control HbA1c in all patients with T2DM. Apart from the need for subcutaneous injection, the two main drawbacks associated with insulin therapy are weight gain and hypoglycemia [6].

The discomfort caused by hypoglycemia in patients with DM is well known, with adverse consequences beyond the acute moment such as the need for admission, therapy readjustments, loss of work or school days, traffic accidents, etc. In addition, repeated hypoglycemia can have a negative impact at the CV and neurological level. Severe hypoglycemia can cause seizures and even coma, but if the hypoglycemia is repeated, it can be associated with cognitive impairment. At the CV level it can induce arrhythmias and even acute coronary events. The available data support its role as a CV risk factor [98]. However, there is controversy as to whether it is a risk marker or whether we should consider hypoglycemia as a CV risk factor as itself.

Traditionally we have focused on improving HbA1c, but we have ignored the fact that it is still an average of blood glucose values. Glycemic variability is a measure of dispersion that reflects variations in glucose, so it is not only important to have an adequate HbA1c, but also that this is achieved with acceptable variability. There are different ways of measuring glycemic variability and currently there is not an ideal method, although the coefficient of variation of glycemia provides a fairly approach. Glycemic variability has been associated with higher levels of oxidative stress and endothelial dysfunction, factors implicated in the development of CV disease. In addition, high glycemic variability might increase the risk of CV events due to a higher rate of severe hypoglycemia [99].

Intensive treatment undoubtedly reduces microvascular complications in both type 1 DM and T2DM. In the DCCT study, intensive treatment with insulin therapy in type 1 DM was associated with a reduction in CV events [100].

The most relevant clinical trial regarding CV risk and insulin was the ORIGIN study [101]. The initial hypothesis of this study was that early insulin glargine U100 use compared to standard treatments would reduce CV events. This study included 12,537 patients with T2DM, impaired glucose tolerance, or impaired fasting glycemia who had already experienced a CV event (59% of patients) or were at high CV risk (41% of patients). The rate of CV events was similar in both arms: 2.94/1000 person-years in the glargine group and 2.85/1000 person-years in the standard treatment arm (HR 1.02; 95% CI 0. 94–1.11). In the insulin treatment group, the number and severity of cases of hypoglycemia, as well as weight, significantly increased. The rate of progression from prediabetes to diabetes was significantly lower in the group receiving insulin. 

The DEVOTE study [102] was designed to assess the CV safety of degludec versus Glargine U100 in patients with T2DM and high CV risk. Degludec was shown to be non-inferior to glargine in terms of three-point MACE (CV mortality, non-fatal stroke, and non-fatal myocardial infarction) (HR 0.91; 95% CI 0.78–1.06). Severe hypoglycemia was significantly less frequent in the degludec group (HR 0.6; 95% CI 0.48–0.76; *p* < 0.001). Similarly, in the degludec group there were fewer severe cases of nocturnal hypoglycemia (HR 0.47; 95% CI 0.31–0.73; *p* < 0.001).

A secondary analysis of this study (DEVOTE-2) [103] showed that less variability in fasting glucose was associated with lower rates of severe hypoglycemia and all-cause mortality. Another secondary analysis of DEVOTE (DEVOTE-3) [104] highlighted the relationship between severe hypoglycemia and all-cause mortality; however, the nature of this relationship was not clarified.

More recently, although the CONCLUDE study [105] was not a CV safety study, it provided data about the risk of hypoglycemia with two basal insulins. The primary endpoint was the rate of hypoglycemia with insulin degludec U200 and insulin glargine U300. In the CONCLUDE trial, 1609 patients with T2DM were randomized to degludec 200 U/mL (degludec U200) or glargine U300. During the maintenance period, HbA1c improved to a similar extent in the two groups with no significant difference in the rate of overall hypoglycemia (the primary study endpoint), while the rates of symptomatic nocturnal and severe hypoglycemia (secondary endpoints) were lower with degludec U200 than with glargine U300. As the primary objective was not met, the secondary analyses were exploratory. In addition, the design of the study had to be modified due to the unreliability of the glucometers initially used in the trial, particularly in the low blood glucose ranges.

With the current data, it can be stated that the use of insulin degludec or Glargine U100 is not associated with an increased CV risk in patients with T2DM and a high-risk profile.

## 4. Metabolic Surgery

Metabolic surgery is very effective in improving glycemic control in patients with T2DM and frequently produces disease remission. The benefits also include a much higher weight reduction than that achieved with non-surgical treatment, as well as a reduction in the number of antihyperglycemic drugs. The results of a recent meta-analysis showed that bariatric surgery in patients with T2DM increased diabetes remission and reduced microvascular and macrovascular complications compared with non-surgical treatment [106]. Metabolic surgery is currently indicated in patients with T2DM with a BMI ≥ 30 kg/m^2^ who have not achieved adequate glycemic control despite lifestyle modification and intensification of treatment with antihyperglycemic drugs.

## 5. Diabetes and CKD. Importance of Estimating Renal Function and Albuminuria in Patients with T2DM

### 5.1. Assessment of Renal Involvement: How?

The degree of CKD and its progression are estimated by determining plasma creatinine and calculating the eGFR (mL/min/1.73 m^2^) and the urine albumin/creatinine ratio (mg/g or mg/mmol). The prognosis and progression of renal involvement are marked by this double dimension. This stratification of the risk of renal involvement is valid for both patients with and without DM, showing some risk categories [107,108].

As most patients with DM receive drugs that block the renin-angiotensin-aldosterone system with the aim of achieving greater cardioprotection and nephroprotection, it is recommended to include serum or plasma potassium determination in the usual analytical controls. This will be helpful to evaluate renal function (through eGFR), the possibility of progression (albuminuria), the dose adjustment of some of the antihyperglycemic treatments, and to estimate the risk of possible development of hyperkalemia [107,108].

### 5.2. What Type of Equation Should Be Use to Estimate GFR?

The estimation of renal function should preferably be carried out using the CKD-EPI equation (formula derived from the Chronic Kidney Disease Epidemiology Collaboration). The results produce less underestimation than the MDRD formula (formula derived from the Modification of Diet in Renal Disease Study) [107,108]. Recently it has been postulated in United States that race should be withdrawn from the CKD-EPI formula, but this has not been validated in Europe [109].

The use of creatinine-derived equations is not appropriate in patients with extreme body weight (BMI < 19 kg/m^2^ or >35 kg/m^2^) or amputations. In these cases, 24-h urine creatinine clearance is required to calculate renal function. There are not enough studies to define the most appropriate formula in patients with obesity and DM, and this can be difficult, especially in patients with DM and a BMI ≥ 30 kg/m^2^, in which the equations derived from creatinine can underestimate renal function to a greater or lesser degree, and this may have consequences in the dosage and prescription of some antihyperglycemic agents [107,108].

### 5.3. Which Is the Best Sample to Estimate Albuminuria?

Albuminuria is estimated using the urine albumin/creatinine ratio in an isolated sample. Any urine sample can be valid, but the first morning sample shows less variability [107,108].

### 5.4. How Often Should Renal Function Be Estimated in Patients with T2DM?

The eGFR, serum creatinine, serum potassium and albuminuria are determined:

- At the time of diagnosis of T2DM.
- At the time of routine controls and, in any case, once a year.
- When starting an antihyperglycemic treatment that may require dosage adjustment.
- If any complication occurs that may involve changes in renal function, or any type of acute complication, comorbidities, or treatments that may temporarily modify renal function (e.g., excessive volume depletion by diuretics, hypotension, vomiting, use of non-steroidal anti-inflammatory drugs, etc.).
- In cases of unexpected hypoglycemic episodes, in the absence of changes in glucose-lowering.
- When insulin requirements are consistently reduced over time (<3 months).
- When starting treatment with SGLT2i, a subsequent evaluation of renal function should be carried out between the first and third month after the initiation of therapy.

### 5.5. Drugs of Choice in Patients with Diabetes and CKD

SGLT2i, as demonstrated in different clinical trials, such as CREDENCE [78], DAPA-CKD [79], EMPA-KIDNEY [80], or SCORED [81] have a positive impact on the progression of CKD in patients with CKD, regardless of the presence of T2DM. However, other drugs have also shown clinical benefits in this clinical setting. 

Traditionally, ACEi and ARB have been used to reduce the progression of CKD, particularly in subjects with T2DM. However, despite their beneficial effects demonstrated in clinical trials, CKD continues to progress despite their use [110,111,112].

On the other hand, the stimulation of the mineralocorticoid receptor is implicated in the etiopathogenesis of CKD. However, although spironolactone and eplerenone, added to ACEi or ARB, reduce proteinuria, no benefit has been shown on the progression of CKD with these drugs [113,114]. In contrast, finerenone, a non-steroidal mineralocorticoid receptor antagonist, has not only been shown to reduce proteinuria, but also to slow the progression of CKD (stages 3 and 4 with albuminuria) and T2DM [115,116].

The FIDELIO-DKD study [115] included 5674 patients with T2DM, a urinary albumin/creatinine ratio of 30–5000 mg/g and eGFR of ≥25 to <75 mL/min/1.73 m^2^, treated with maximum tolerated doses of renin-angiotensin system inhibitors. Patients were randomized to receive finerenone or a placebo. The primary endpoint, assessed in a time-to-event analysis, was renal failure, a sustained decrease of at least 40% in the eGFR from baseline, or death from renal causes. 45.9% of patients had a history of CV disease. After a follow-up of 2.6 years, finerenone reduced the risk of the primary composite endpoint by 18% compared with the placebo (HR 0.82; 95% CI 0.73–0.93; *p* = 0.001), regardless of the history of prior CV disease.

FIGARO-DKD [116] was a clinical study evaluating finerenone in 7437 patients with CKD (eGFR 25–90 mL/min/1.73 m^2^ and urinary albumin excretion 30 -< 300 mg/g or patients with urinary albumin excretion 300–5000 mg/g and eGFR ≥ 60 mL/min/1.73 m^2^) and T2DM. All participants were treated with ACEi or ARB at the maximum tolerated doses. The primary endpoint of the study was a cardiac composite of CV death, nonfatal acute myocardial infarction, nonfatal stroke, or HF hospitalization. The first secondary endpoint was a renal composite of end-stage renal disease, a sustained decrease in eGFR of at least 40% compared with baseline, or death from renal causes. After a median follow-up of 3.4 years, 12.4% in the finerenone group and 14.2% in the placebo group presented the main objective of the study (HR 0.87; 95% CI 0.76–0.98; *p* = 0.03), the benefit being higher for HF hospitalizations (HR 0.71; 95% CI 0.56–0.90). Regarding the secondary endpoint, this occurred in 9.5% of the finerenone group and in 10.8% of the placebo group (HR 0.87; 95% CI 0.76–101). The incidence of adverse events was similar in the two groups. Discontinuation of the study due to hyperkalemia was higher in the finerenone group than in the placebo group (1.2% vs. 0.4%).

## 6. Treatment Proposal

The following management proposal for patients with high or very high CV risk, HF or diabetic CKD, from the Diabetes and Obesity Working Group of the Spanish Society of Cardiology, has been made from a multidisciplinary approach with the participation of endocrinologists, internists, general practitioners, nephrologists and cardiologists.

This proposal was first published in 2019 [117] and was updated in 2020 [118] due to the publication of new positive evidence.

**Figure 1:**
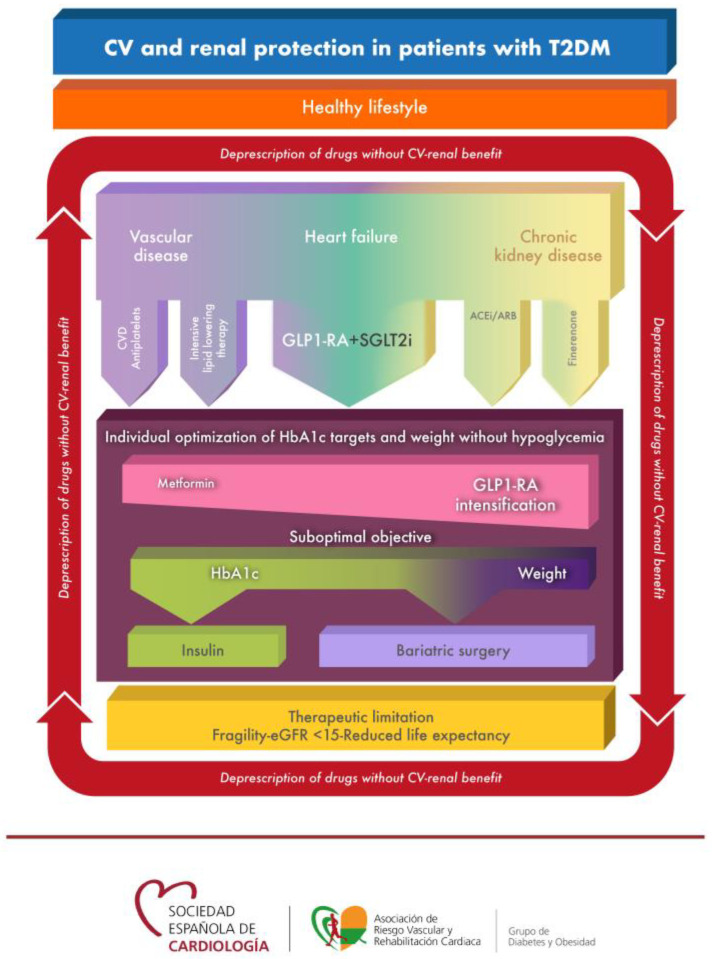
Therapeutic recommendations for CV and renal protection in patients with T2DM. ACEi: Angiotensin-converting enzyme inhibitors; ARB: angiotensin receptor II blockers; CV: cardiovascular; eGFR: estimated glomerular filtration rate; GLP1-RA: glucagon-like peptide receptor agonists; SGLT2i: sodium-glucose cotransporter type 2 inhibitors; T2D: type 2 diabetes.

The therapeutic algorithm presented in Figure 1 is based on the CV safety and efficacy results of the drugs assessed in the randomized clinical trials published to date, as well as on the characteristics of the patients included in these studies.

There are drugs that do not appear in the algorithm because they have not demonstrated CV safety or efficacy; they are currently being evaluated in ongoing studies, or they have not shown a CV benefit, or even an increased risk of HF, such as pioglitazone or some DPP4i.

Particular attention is paid in this consensus statement to the awareness of the continuous risk of developing HF, CKD and atherosclerosis that leads to MACE (CV death, non-fatal myocardial infarction or non-fatal stroke), hospitalization for unstable angina or need for new revascularization. This risk is minimized by drugs that are placed in the first therapeutic step through different mechanisms of action and with positive results in clinical trials; some of them act mainly on the prevention and treatment of HF or CKD, and others on the prevention of new atherothrombotic events. As Figure 1 shows, the risk of these complications does not disappear over time, highlighting that any patients with T2DM can develop these complications during the course of the disease.

It is also highlighted that metabolic control does not only consist of HbA1c control. Therefore, weight reduction and decrease in risk of hypoglycemia are also included as an essential part of this control. 

On the other hand, the comprehensive management of patients with T2DM and CV disease is challenging due to the great number of comorbidities and treatments. It is important to assure that these patients take those drugs that have demonstrated CV benefit. In this context, the development of effective multidisciplinary teams would be very helpful in order to provide a comprehensive management of this population [119]. Remarkably, despite the evidence provided by clinical trials demonstrating the CV benefits of treatment with some drugs, there are some potential barriers and challenges that may limit their implementation in clinical practice. These reasons may include scarce knowledge or awareness by health care providers, insufficient communication and integration between health care levels, lack of incentives, etc. As a result, to actually reduce these gaps, it is important to enhance medical education and improve communication between all health care providers [120,121].

Based on all this, the following recommendations are made:

### 6.1. Definition of Metabolic Control

#### 6.1.1. Recommendation

The definition of metabolic control includes a combined goal of glycemic control, weight reduction, and no hypoglycemia. The specific objectives are:

(a) Glycemic control:
  - HbA1c < 5.7% in patients without prior pharmacological treatment, or with a short evolution of DM, with the aim of reaching normoglycemia with the combination of GLP-1 receptor agonists/SGLT2i.
  - HbA1c ≤ 6.5% in patients with more advanced disease, if it can be achieved with drugs that do not induce hypoglycemia or weight gain.
  - HbA1c ≤ 7% (or higher) in patients at high risk of severe hypoglycemia, frail patients, or patients with limited life expectancy.
  - In patients using continuous glucose monitoring systems, the glucose management indicator target (previously called estimated HbA1c) will be the same as that of the laboratory HbA1c, maintaining a time in range greater than 80% (if target HbA1c < 6.5%) or higher than 70% (if target HbA1c < 7%), as well as low glycemic variability (coefficient of variation ≤ 36%).
(b) Avoid hypoglycemia:
  - Do not use SU, glinides or rapid insulin and limit basal insulin to patients who do not achieve adequate glycemic control despite intensification with cardioprotective antihyperglycemic drugs.
  - In patients receiving insulin treatment, the use of continuous glucose monitoring is recommended (with the activation of hypoglycemia alarms) and to achieve a time in hypoglycemia <70 mg/dL less than 4% and a time in hypoglycemia <55 mg/dL less than 1%.
(c) Weight loss:
  - The general objective is to promote a weight loss of at least 10% in 1 year in patients with a BMI ≥ 25 kg/m^2^ or increased abdominal circumference (≥102 cm in men and ≥88 cm in women), at the expense of ectopic fat deposition and preserving muscle mass. Weight loss can also contribute to achieving other general patient goals such as LDLc or blood pressure control.
  - In patients with a short time of evolution of T2DM, losses ≥15% can achieve remission of diabetes.
  - In all other patients (except cases of frailty) it is recommended to avoid weight gain.

#### 6.1.2. Support of the Recommendation

Intensive glycemic control reduces the microvascular complications of T2DM, but data on CV risk reduction are less clear [122]. Results of a meta-analysis showed that intensive glycemic control reduced the risk of non-fatal myocardial infarction by 17%, but with no benefits in CV mortality, all-cause mortality, stroke, or HF [123]. In fact, in the ACCORD study [124], there was an increase in all-cause mortality. In all these studies, drugs that favored hypoglycemia and weight gain were used. Severe hypoglycemia has been associated in multiple trials with increased CV morbidity and mortality and all-cause mortality, so it is a priority to avoid drugs that induce hypoglycemia in patients with T2DM and high CV risk [104,125].

Various publications have shown that a HbA1c level of 6.5% does not clearly define the risk zone for developing complications of T2DM. A recent example is a meta-analysis that included 129 studies with a total of 10 million people with prediabetes (i.e., fasting plasma glucose between 100 and 125 mg/dL, HbA1c between 5.7 and 6.4 % or glycemia after oral glucose overload between 140 and 199 mg/dL). Despite not having an HbA1c greater than 6.5% or a fasting plasma glucose greater than 125 mg/dL, these patients presented an increased CV risk. Specifically, prediabetes defined as fasting plasma glucose between 100–125 mg/dL was associated, compared with normoglycemia, with an increased risk of CV disease, stroke, and mortality, and prediabetes defined as HbA1c between 5.7 and 6.4% was associated with an increased risk of CV disease compared with people who had HbA1c in the normal range [126].

At present, weight loss of more than 10–15% with lifestyle modification together with GLP-1 receptor agonist/SGLT2i allows the attaining of HbA1c < 5.7% in patients with short-term T2DM and maintained pancreatic reserve, so a goal of normoglycemia could be reached. In patients with a longer evolution of DM, similar to the AACE (American Association of Clinical Endocrinologists) consensus [127], we recommend an HbA1c target of ≤6.5% if it can be achieved with drugs that do not induce hypoglycemia or weight gain; otherwise, the target is raised to HbA1c ≤ 7% (or higher in the case of limited life expectancy, severe hypoglycemia, frailty, or multiple associated comorbidities that make it difficult to achieve an adequate glycemic control).

The increasing use of interstitial glucose monitoring, in its real-time continuous monitoring (CGM) and on-demand or flash glucose monitoring (FGM) modalities, has led to a change in the model in the management of patients with DM, mostly in type 1 DM but progressively spreading to patients with T2DM on insulin treatment. According to the new concept of glycemic control, it is not enough to achieve the HbA1c target, but also to do so with low variability and low risk of hypoglycemia. The CGM/FGM allows the analysis of the past by studying the glycemic parameters and the ambulatory glucose profile, to evaluate the present with the instantaneous determination of interstitial glucose, and to make therapeutic decisions regarding the immediate future thanks to the trend arrows. The appropriate use of CGM/FGM can improve glycemic control and reduce the risk of hypoglycemia in patients with DM, by incorporating hyper/hypoglycemia alarms. The international consensus on time in range recommends a target range between 70 and 180 mg/dL in patients with T2DM, and maintaining this range for >70% of the time, which would be equivalent to an HbA1c < 7% [128]. If the target HbA1c is <6.5% the target time in range should be >80%. This objective must also be accompanied by a low incidence of hypoglycemia (time in hypoglycemia <70 mg/dL less than 4%, time in hypoglycemia <55 mg/dL less than 1%) and low glycemic variability (coefficient of variation < 36%).

The risk of T2DM and CV disease increases exponentially as the BMI rises above 25 kg/m^2^ in the Caucasian population and the waist circumference increases above 94 cm in men and 80 cm in women, although the risk is considered very high with a waist circumference ≥102 cm in men and 88 cm in women [129]. Excess weight is common in individuals with T2DM, and it is estimated that 50% and 80% have a BMI ≥ 30 kg/m^2^ and 25 kg/m^2^, respectively [130]. According to the data from the Di@bet.es study, the prevalence of obesity and abdominal obesity in Spaniards with T2DM reaches 50% and 68%, respectively [2].

Weight loss through caloric restriction, whether with lifestyle modification, drugs, or bariatric surgery, reduces intrahepatic fat, insulin resistance, hepatic glucose production, and circulating triglycerides and their accumulation in pancreatic islets. The resolution of pancreatic steatosis favors the redifferentiation of beta cells, which were not dead but dedifferentiated by the accumulation of fat, and this translates into a normalization of endocrine pancreatic function. This effect reaches its maximum impact when the weight loss is greater than 15% of the basal weight [131].

Lower weight losses (5–15%) reduce glucolipotoxicity and thus improve insulin sensitivity, glycemic control, blood pressure, and lipid profile, and decrease the need for antihyperglycemic drugs [130,131]. In the DIRECT study [14], that was carried out in individuals with T2DM of less than 6 years of evolution without insulin therapy and BMI 27–45 kg/m^2^, an intensive intervention through caloric restriction, compared with a standard management of T2DM, showed a remission of the disease, without requiring pharmacological treatment, in 46% of patients in the intervention group compared to 4% in the control group (*p* < 0.0001). This percentage of remission increased with greater weight loss, reaching 86% in patients who had lost >15 kg. At present, we have potent GLP-1 receptor agonists and SGLT2i whose combination at high doses together with lifestyle modification can facilitate the goal of weight loss. The upcoming marketing of GLP-1/GIP dual agonists, such as tirzepatide will increase the probability of achieving weight losses >10–15%.

In a sub-analysis of the intensive weight intervention study LOOK-AHEAD [132] (whose overall result was neutral), those patients with T2DM who lost more than 10% of their baseline weight in the first year had a 21% reduction in CV morbidity and mortality (MACE 4) compared to patients who did not lose weight. In a meta-analysis, bariatric/metabolic surgery in patients with T2DM increased diabetes remission and reduced microvascular and macrovascular complications compared with non-surgical treatment [106]. Several randomized controlled clinical trials with usual medical treatment have shown a greater probability of remission of T2DM with bariatric/metabolic surgery. In one of the most relevant studies, 95% of patients with biliopancreatic diversion and 75% of patients with gastric bypass achieved disease remission vs. 0% in the medical treatment group. At 10 years these percentages had been reduced to 50% in the biliopancreatic diversion group and 25% in the gastric bypass group [133]. These data and others, such as those of the SOS study in Sweden after 15 years of follow-up, point to a long-term remission in around a third of patients undergoing bariatric/metabolic surgery [134].

Although the definition of remission of T2DM implies the withdrawal of all antihyperglycemic drugs, the ethical dilemma of discontinuing those therapeutic classes with CV and renal benefits, such as GLP-1 receptor agonists and SGLT2i, arises in patients with high cardio-renal risk. Certain molecules also have an indication in people without T2DM, such as some GLP-1 receptor agonists in obesity or some SGLT2i in HF and CKD. Therefore, although their withdrawal would maintain disease remission in some patients, we would nevertheless be depriving them of proven benefits on their morbidity and mortality, which is why we recommend maintaining these drugs despite reaching normoglycemia. In order to reach an intermediate meeting point in this debate, we have collected from the literature an alternative definition of “remission of T2DM induced by drugs” or “drug-sensitive”, in which individuals would maintain normoglycemia under antihyperglycemic treatments that mimic the caloric restriction, such as GLP-1 receptor agonists and SGLT2i, and drugs for obesity [135].

### 6.2. Explanation of Optimization of Metabolic Control

#### 6.2.1. Recommendation

- Start (first assessment)

Scenario 1 (naïve patient): The combination of GLP-1 receptor agonists/SGLT2i will be prescribed from the beginning, regardless of HbA1c, selecting from each group those drugs that have demonstrated CV and renal benefit. Neither the indication nor the benefits of GLP-1 receptor agonists are limited by the patient’s BMI, although their reimbursement is limited by the payers in some countries. In case of contraindication for any of these two therapeutic groups, metformin will be associated.

Scenario 2 (patient with previous treatment that does not include GLP-1 receptor agonists or SGLT2i): Metformin will be maintained if the patient was already taking it and withdrawal (or at least dose reduction) of those drugs without proven CV benefit will be attempted (SU, glinides, DPP4i, insulin), replacing them with the combination of GLP-1 receptor agonists and SGLT2i. Pioglitazone should be discontinued in patients at risk of HF.

- Intensification (after the introduction of GLP-1 receptor agonists/SGLT2i)

Scenario 1: (patient HbA1c ≤ 1.0% above target or insufficient weight loss): Intensify treatment with high-dose GLP-1 receptor agonists (semaglutide 2.0–2.4 mg or dulaglutide 3.0–4.5 mg) or switch to a high potency GLP-1 receptor agonist (in case of lixisenatide, liraglutide, exenatide, or standard dose of dulaglutide). The switch of SGLT-2i to high doses of canagliflozin (300 mg) could also be considered.

Scenario 2: (patient HbA1c > 1.0% above target): changes recommended in scenario 1 will be carried out and metformin will also be associated.

In patients with contraindications or intolerance to GLP-1 receptor agonists, or in those individuals with reimbursement restrictions who cannot afford treatment, DPP4i can be used, given their neutral effect on CV events, weight, and hypoglycemia. 

#### 6.2.2. Support of the Recommendation

Several consensus documents from different scientific societies currently recommend prescribing a GLP-1 receptor agonist and/or an SGLT-2i with demonstrated CV benefit in patients with high CV risk or established CV or renal disease, regardless of HbA1c levels, and before the prescription of metformin, since the cardiorenal protection of these drugs is independent of the degree of glycemic control or concomitant treatment with metformin [3,5]. In this document, we recommend starting with the combination of GLP-1 receptor agonists/SGLT2i, given that the CV and renal benefits provided by both groups are different and probably additive or synergistic [136,137], their concomitant use is safe and achieves long-term sustained metabolic control [138]. If the patient was already taking other antihyperglycemic drugs without CV or renal benefit, we recommend deprescribing these drugs (see below).

In patients with suboptimal metabolic control after 3 months of treatment, therapy will be intensified. If the patient’s HbA1c is close to target (≤1.0% above target) or weight loss has been insufficient, it is recommended to intensify treatment with GLP-1 receptor agonists, increasing to high doses (semaglutide 2.0–2.4 mg or dulaglutide 3.0–4.5 mg) [139,140] or switch to a high-potency GLP-1 receptor agonist if the patient is taking liraglutide, lixisenatide, exenatide, or standard-dose dulaglutide [141]. Switching from an SGLT2i to high-dose canagliflozin might also be considered (300 mg) given its greater efficacy in reducing HbA1c, weight, and blood pressure, although this recommendation is only supported by observational studies [142]. In those patients with suboptimal glycemic control and HbA1c > 1.0% above target, it is recommended to intensify GLP-1 receptor agonist therapy and add metformin simultaneously.

Several GLP-1 receptor agonists (liraglutide, semaglutide, albiglutide, dulaglutide, efpeglenatide) and SGLT2i (empagliflozin, canagliflozin, dapagliflozin, sotagliflozin) have shown CV and renal benefit in patients with T2DM and high CV risk [143]. A recent meta -analysis concludes that GLP-1 receptor agonists significantly reduce MACE3 by 14%, CV death by 13%, all-cause mortality by 12%, myocardial infarction by 10%, stroke by 17%, admissions for HF by 11%, and CKD progression by 21% [143]. Regarding SGLT2i, two meta-analyses have shown significant reductions of 12% in MACE, 15% in CV death, 14% in all-cause mortality, 11% in myocardial infarction, 32% in hospitalizations for HF, and 36% in CKD progression, but not in strokes [144,145].

Metformin showed a reduction in CV morbidity and mortality in a small group of overweight patients (*n* = 342) in the UKPDS study compared to diet or SU or first-generation insulins, a result that would be insufficient for approval by any regulatory agency at this moment [47]. In fact, a meta-analysis of randomized clinical trials that included the UKPDS concluded that metformin has a neutral effect on the incidence of myocardial infarction and CV or all-cause morbidity and mortality [48]. On the other hand, although in studies of CV safety with GLP-1 receptor agonists or SGLT2i 75% of patients were on treatment with metformin, the CV and renal benefit was also seen in the subgroup of patients that were not taking this drug, suggesting that the cardio- and nephroprotective effect of these drugs was independent of concomitant treatment with metformin [146,147,148].

Regarding pioglitazone, although in the PROACTIVE study the secondary objective MACE3 was significantly reduced by 16% in patients with DM and CV disease, the increased risk of HF and weight gain of this drug implies that it does not have a favorable profile in patients with CV disease, at least with the 45 mg dose used in this study. It is unknown whether lower doses can maintain the CV benefit without increasing the risk of HF [63].

### 6.3. Explanation of De-Intensification

#### 6.3.1. Recommendation

- If the patient has an optimal glycemic control but is receiving non-cardioprotective antihyperglycemic therapy, drugs without CV benefit, such as SU, glinides or DPP4i, will be replaced by cardioprotective drugs.
- In those patients on insulin treatment, complex regimens will be simplified by switching to basal insulin and the daily dose of insulin will be reduced, in order to reduce the risk of hypoglycemia and weight gain.

#### 6.3.2. Support of the Recommendation

If the patient has an optimal glycemic control but is receiving non-cardioprotective antihyperglycemic therapy, drugs without CV benefit, such as SU, glinides or DPP4i, will be replaced by cardioprotective drugs. In patients treated with insulin and optimal control, a de-intensification strategy will be considered; complex bolus/basal insulin therapy regimens or biphasic mixtures will be simplified by stopping prandial insulin and prescribing a basal insulin that has demonstrated CV safety. In those patients who only receive basal insulin therapy, the previous dose will be reduced by 20–30%, adjusting the dose based on fasting capillary blood glucose controls. In some patients it is possible to completely discontinue insulin, although this is not a general objective, as this depends on individual patient characteristics, such as the reduction in insulin resistance induced by weight loss or surrogate markers of pancreatic function, such as the time of evolution of DM, years of insulin therapy, C-peptide levels, or the presence of autoantibodies against beta cells [149].

The AWARD-7 study shows that the addition of dulaglutide to the administration of insulin glargine in patients with CKD and T2DM was similar to basal-bolus treatment in metabolic control and superior in renal benefits. In all studies in which a GLP-1 receptor agonist is added to a basal insulin, the basal insulin dose is reduced [150]. In the SUSTAIN 5 study with semaglutide, the basal insulin dose was reduced by 20% before adding the GLP-1 receptor agonist [151]. By adding subcutaneous semaglutide to basal insulin therapy, the insulin dose decreased and the largest decreases in insulin were seen in patients with baseline HbA1c < 8.0%. Recently, the SPARE study [152] was a real-life study carried out in endocrinology clinics in Canada that included patients who started with GLP-1 receptor agonists added to their usual treatment. Patients on basal-bolus insulin required a 20% lower mean total insulin dose after adding subcutaneous semaglutide, and those on basal insulin alone reduced their doses by 10% (both significantly). 

When choosing a basal insulin, it is essential not only to consider aspects such as glycemic variability and the risk of hypoglycemia, but also CV risk. In patients with established CV disease, CKD or high CV risk, an insulin with evidence of CV safety and low risk of hypoglycemia should be chosen (degludec and glargine insulin).

In patients with T2DM and obesity, treated with basal-bolus regimens or two or three daily doses of premixed insulin, and who require very high doses of insulin, with weight gain, hypoglycemia, or poor glycemic control, a simplified protocol of the insulin therapy regimen could be considered, similar to that proposed by Naing et al. [153] This change would allow the simplification of insulin treatment in those individuals with complex regimens, substituting prandial insulin for GLP-1 receptor agonists. This study showed that, in patients with obesity with poor glycemic control (HbA1c > 8%) treated with a basal-bolus regimen or two or three doses of fixed mixtures, a simplified regimen that included basal insulin, a GLP-1 receptor agonist, metformin and an SGLT2i, allowed the achievement of a significant reduction in HbA1c, weight, total insulin dose and an improved treatment satisfaction index, when compared with intensification with multiple doses of insulin. The proposed treatment regimen was as follows:

- Withdrawal of all rapid insulin.
- Decrease basal insulin by 20%.
- Metformin maintenance.
- In patients with two or three fixed mixtures, replacement by a single dose of basal insulin, in an amount equivalent to 40% of the total dose of the mixture.
- Addition of an SGLT2i.
- Addition of a GLP-1 receptor agonist, with progressive dose escalation.

A review of how to deprescribe or de-intensify insulin therapy with the addition of SGLT2i and/or GLP-1 receptor agonists in patients with T2D and CKD has recently been published [154].

### 6.4. Explanation of the Choice of Drugs in Patients with eGFR < 15 mL/min

- In patients with eGFR < 15 mL/min/1.73 m^2^ on treatment with canagliflozin 100 mg, empagliflozin 10 mg or dapagliflozin 10 mg, maintain treatment until dialysis, given the CV and renal benefits.

Some antihyperglycemic agents such as SU and insulin are eliminated by the kidneys, so it is necessary to adjust the dose to avoid the appearance of hypoglycemia. DPP4i have different degrees of renal elimination, so it is necessary to adjust the dose based on pharmacokinetic studies. However, no risk of hypoglycemia with reduced eGFR has been described when used alone or with drugs that are not eliminated by the kidneys. In the case of GLP-1 receptor agonists, there are drugs with renal elimination (exenatide, lixisenatide) that require dose adjustment [152]. Dose limitation of GLP-1 receptor agonists up to 15 mL/min/1.73 m^2^ is based solely on the lack of studies since the pharmacokinetic studies of liraglutide, semaglutide and dulaglutide show that the area under the curve of these drugs is similar in patients on dialysis and in people with normal renal function [155,156].

In the case of SGLT2i, the reduction in glomerular filtration implies a lower hypoglycemic effect. In fact, the summary of product characteristics of SGLT2i indicate that when SGLT2i are administered in patients with eGFR < 45 mL/min/1.73 m^2^, it is recommended to consider additional glucose-lowering treatment. The latest evidence on the CV and renal benefit of SGLT2i has led to continuous changes in the summary of product characteristics and in international consensus regarding glomerular filtration thresholds for the initiation and maintenance of treatment with SGLT2i. The ADA/EASD recommendations indicate that SGLT2i can be initiated in patients with eGFR rate >20 mL/min/1.73 m^2^ [122]. The current indications according to the eGFR of each SGLT2i are indicated below:

- Dapagliflozin: Dose of 10 mg/day. Initiate dapagliflozin up to 25 mL/min/1.73 m^2^, which can be maintained until the start of dialysis. Dapagliflozin can also be used for CKD or HF, regardless of T2DM status.
- Empagliflozin: In T2DM, 10 mg/day, which can be increased to 25 mg/day if tighter glycemic control is required. In HF the dose is 10 mg/day. Empagliflozin can be used in patients with T2DM and CV disease, up to eGFR of 30 mL/min/1.73 m^2^; and up to 20 mL/min/1.73 m^2^ in case of HF.
- Canagliflozin: In T2DM, dose of 100 mg/day. In patients with eGFR > 60 mL/min/1.73 m^2^ and if greater glycemic control is required, it can be increased to 300 mg/day. For the treatment of diabetic CKD, the dose is 100 mg/day. Treatment can be maintained up to eGFR of 30 mL/min/1.73 m^2^. If the patient has eGFR < 30 mL/min/1.73 m^2^ and a urine albumin/creatinine ratio >300 mg/g, it can be maintained until the start of dialysis or renal transplantation.
- Ertugliflozin: Dose of 5 mg/day. It can be increased to 15 mg/day when greater glycemic control is needed. It is not recommended to start ertugliflozin in patients with eGFR < 45 mL/min/1.73 m^2^. Interrupt treatment in case of eGFR < 30 mL/min/1.73 m^2^.

#### 6.4.1. Explanation of Choice of GLP-1 Receptor Agonists

##### Recommendations

- According to EMA regulations and the summary of product characteristics, semaglutide, dulaglutide and liraglutide can be administered up to 15 mL/min/1.73 m^2^; exenatide, and lixisenatide up to 30 mL/min/1.73 m^2^ [157].
- In patients with eGFR < 15 mL/mL/1.73 m^2^ on previous treatment with a GLP-1 receptor agonist and well tolerated, consider maintaining it given their CV benefits.
- In case of intolerance to GLP-1 receptor agonists with drop in glomerular filtration, prescribe a DPP4i.

##### Support of the Recommendation

In patients with stage 5 CKD, we have few therapeutic alternatives except insulin and DPP4i. Although there are limited data with GLP-1 receptor agonists in patients with stage 5 CKD [158], their initiation is currently not indicated in this group of patients. However, given the CV benefits and the low risk of hypoglycemia, if the patient was previously receiving treatment with a human GLP-1 analogue (which is not eliminated by the kidneys), we recommend not discontinuing it. Within SGLT2i, canagliflozin (100 mg) and dapagliflozin (10 mg) currently have an indication to maintain their treatment until dialysis [159]. In fact, different international guidelines recommend maintaining the treatment with SGLT2i until dialysis [3,6,108]. 

DPP4i represent a pharmacological class that can be used (with dose adjustment, except for linagliptin, which does not require it) in patients with stage 5 CKD, including dialysis, meeting the criteria for CV safety and having a neutral effect on weight and hypoglycemia.

### 6.5. Explanation of Choice of Insulin

#### 6.5.1. Recommendation

In patients with T2DM and CV disease or high CV risk who are not controlled after intensification to triple therapy with cardioprotective drugs, start basal insulin therapy with insulin degludec, given the proven CV safety compared to insulin glargine U100, with a significant reduction in the risk of severe total and nocturnal hypoglycemia. 

#### 6.5.2. Support of the Recommendation

Two insulins have CV safety studies in patients with T2DM and high CV risk: glargine U100 and degludec. In the ORIGIN study [101], glargine U100 showed non-inferiority in MACE3 or MACE5 compared to conventional antihyperglycemic treatment that did not initially include insulin, although it increased the risk of total and severe hypoglycemia. In the DEVOTE study [102], insulin degludec showed non-inferiority in MACE3 versus glargine U100, but reduced the risk of severe hypoglycemia by 40% and severe nocturnal hypoglycemia by 53%. As an exploratory finding that was not part of the main analysis of the study, the subgroup of women showed a significant reduction in MACE3 of 24% with degludec. Insulin glargine U300 has not had a CV safety study. Although the regulatory agencies have not requested such a study, the truth is that glargine U300 is an insulin with different pharmacokinetic and pharmacodynamic characteristics from glargine U100, so both insulins cannot be considered equivalent from a metabolic or CV point of view. That is why we recommend using insulin degludec as the insulin of choice in patients with T2DM and CVD or high CV risk.

### 6.6. Explanation of the Indication for Bariatric/Metabolic Surgery

#### 6.6.1. Recommendation

Bariatric/metabolic surgery is recommended in patients with BMI ≥ 30 kg/m^2^ who have not achieved adequate glycemic control (metabolic surgery) or weight loss (bariatric surgery) despite lifestyle modification and intensification with antihyperglycemic drugs that induce weight loss or drugs for obesity.

#### 6.6.2. Support of the Recommendation

In Spain, two drugs are currently marketed for the treatment of obesity: orlistat and liraglutide 3 mg. The EMA has authorized the marketing of semaglutide 2.4 mg. All of them are more likely to achieve weight loss >10% than with a placebo, and also significantly reduce HbA1c in patients with T2DM, semaglutide 2.4 mg being the most effective drug of them, with mean weight loss greater than 15 %. Orlistat does not have a CV safety study, although it induces improvement in several CV risk factors in patients with obesity and T2DM [160]. Liraglutide 3 mg has no CV safety study, although the summary of product characteristics has included the favorable data from the LEADER study carried out with the 1.8 mg dose [91]. Semaglutide 2.4 mg is being analyzed in the ongoing SELECT study (patients without T2DM with CV disease) and we also have the favorable results of the SUSTAIN 6 study in patients with T2DM with a dose of 1 mg [92]. In the near future we will have tirzepatide, a dual GLP-1/GIP agonist with a marked effect on weight in patients with T2DM. The SURPASS-CVOT (NCT04255433) study is currently analyzing the CV safety of tirzepatide compared with dulaglutide among patients with T2DM, confirmed atherosclerotic CV disease, HbA1c ≥ 7.0% to ≤10.5% and BMI ≥ 25 kg/m^2^. In addition, the SURMOUNT MMO (NCT05556512) study is determining the effect of tirzepatide on the reduction in morbidity and mortality in adults with obesity (BMI ≥ 27.0 Kg/m^2^) and established CV disease, but without DM.

As explained in previous sections, bariatric/metabolic surgery is very effective in improving glycemic control in patients with T2DM and frequently produces disease remission. The effects can be maintained for at least 5 years. The benefits also include a weight reduction much greater than that achieved by non-surgical treatment, as well as a reduction in the number of antihyperglycemic drugs [161]. Bariatric/metabolic surgery in patients with T2DM increases diabetes remission and reduces micro- and macrovascular complications compared to non-surgical treatment. In 2016, an international consensus conference of six international DM organizations recommended bariatric/metabolic treatment in patients with BMI ≥ 30 kg/m^2^ who have not achieved adequate glycemic control despite lifestyle modification and intensification with antihyperglycemic drugs [162]. Validated techniques such as gastric bypass, tube gastrectomy or biliopancreatic bypass are recommended.

## 7. Conclusions

This document presents a series of recommendations on the comprehensive and simultaneous management of the patient with T2DM, performed by the different specialists that treat these patients (endocrinologists, primary care physicians, internists, nephrologists and cardiologists). In this context, special emphasis is focused on the global control of CV risk factors, the inclusion of weight within the therapeutic objectives, the deprescription of those drugs without CV benefit, and the inclusion of GLP-1 receptor agonists and SGLT2i as protective drugs at the CV level, at the same level as statins, acetylsalicylic acid, ACEi or ARB. In addition, metformin should not be considered as the first therapeutic step as antihyperglycemic treatment in T2DM, but GLP-1 receptor agonists and SGLT2i should be used instead. Likewise, the education of the patient is also considered essential so that the patient can be responsible for the control and evolution of his/her disease.

## Figures and Tables

**Table 1:** Cardiovascular risk factors control targets in subjects with T2DM.

Cardiovascular Risk Factor	Action			
**Dietetic pattern**	Mediterranean diet			
**Smoking**	Stop smoking			
**Physical activity**	>150 min a week of aerobic physical activity + resistance exercise twice a week			
**HbA1c**	≤7.0%, ≤6.5% if possible without hypoglycemia (individualization)			
**Weight**	Reduction in body weight ≥10% if BMI ≥ 25 Kg/m^2^ or waist circumference at risk			
**Lipids**		**Primary objective**	**Secondary objectives**	
	**CV risk**	**LDLc**	**Non-HDL cholesterol**	**ApoB**
	**Moderate**	<100 mg/dL	<130 mg/dL	<100 mg/dL
	**High**	<70 mg/dL and a reduction ≥50% from baseline levels	<100 mg/dL	<80 mg/dL
	**Very high**	<55 mg/dL and a reduction ≥50% from baseline levels	<85 mg/dL	<65 mg/dL
**Blood pressure**	• Systolic: 120 -< 130 mmHg • Diastolic: 70 -< 80 mmHg			

BMI: body mass index; CV: cardiovascular; LDLc: LDL cholesterol; T2DM: type 2 diabetes mellitus. Table performed with data from references [3,5,13].

**Table 2:** Clinical trials showing the CV benefits of GLP-1 receptor agonists and SGLT2 inhibitors in patients with T2DM.

Drugs with CV Benefit in Specific Study Populations	Clinical Trial	Primary and Secondary Endpoints with a Significant Risk Reduction	
		Major CV Events †	HF Hospitalization
**Established CVD**			
**GLP-1 receptor agonists**			
Liraglutide	Liraglutide Effect and Action in Diabetes: Evaluation of Cardiovascular Outcome Results (LEADER) [89]	Primary endpoint ‡	
Semaglutide	Trial to Evaluate Cardiovascular and Other Long-term Outcomes with Semaglutide in Subjects with Type 2 Diabetes (SUSTAIN-6) [90]	Primary endpoint ‡	
Dulaglutide	Researching Cardiovascular Events with a Weekly Incretin in Diabetes (REWIND) [94]	Primary endpoint ‡	
**SGLT2 inhibitors**			
Empagliflozin	Empagliflozin Cardiovascular Outcome Event Trial in Type 2 Diabetes Mellitus Patients (EMPA-REG) [72]	Primary endpoint ‡	Secondary endpoint
Canagliflozin	Canagliflozin Cardiovascular Assessment Study (CANVAS) [74]	Primary endpoint ‡	Secondary endpoint
Dapagliflozin	Dapagliflozin Effect on Cardiovascular Events–Thrombolysis in Myocardial Infarction 58 (DECLARE-TIMI 58) [75]		Primary endpoint ‡
Ertugliflozin	Evaluation of Ertugliflozin Efficacy and Safety Cardiovascular Outcomes Trial (VERTIS CV) [76]		Secondary endpoint
**Multiple CV risk factors**			
GLP-1 receptor agonist, dulaglutide	Researching Cardiovascular Events with a Weekly Incretin in Diabetes (REWIND) [94]	Primary endpoint ‡	
SGLT2 inhibitor, dapagliflozin	Dapagliflozin Effect on Cardiovascular Events–Thrombolysis in Myocardial Infarction 58 (DECLARE-TIMI 58) [75]		Primary endpoint ‡
**Heart failure with reduced ejection fraction** **ǁ**			
**SGLT2 inhibitors**			
Dapagliflozin	Dapagliflozin and Prevention of Adverse Outcomes in Heart Failure (DAPA-HF) [81] ‡		Primary endpoint ‡
Empagliflozin	Empagliflozin Outcome Trial in Patients with Chronic Heart Failure and a Reduced Ejection Fraction (EMPEROR-Reduced) [82]		Primary endpoint ‡
Chronic kidney disease with albuminuria **			
**SGLT2 inhibitors**			
Canagliflozin	Canagliflozin and Renal Events in Diabetes with Established Nephropathy Clinical Evaluation (CREDENCE) [77]	Secondary endpoint	Secondary endpoint ‡
Dapagliflozin	Dapagliflozin and Prevention of Adverse Outcomes in Chronic Kidney Disease (DAPA-CKD) [78]	Secondary endpoint	Secondary endpoint
**Heart failure with preserved ejection fraction**			
Empagliflozin	EMPEROR PRESERVED (EMPagliflozin outcomE tRial in Patients with chrOnic heaRt Failure with Preserved Ejection Fraction) [85]		Primary endpoint
Dapagliflozin	DELIVER (Dapagliflozin Evaluation to Improve the LIVEs of Patients with PReserved Ejection Fraction Heart Failure) [86]		Primary endpoint
**Acute heart failure**			
Empagliflozin	EMPULSE (A Study to Test the Effect of Empagliflozin in Patients Who Are in Hospital for Acute Heart Failure) [84]		Primary endpoint
Dapagliflozin	DAPA-HF (Efficacy and Safety of Dapagliflozin in Acute Heart Failure) [81]		Primary endpoint
Sotagliflozin	SOLOIST-WHF (Effect of Sotagliflozin on Cardiovascular Events in Patients with Type 2 Diabetes Post Worsening Heart Failure) [83]		
**Chronic kidney disease with or without albuminuria**			
Sotagliflozin	SCORED (Effect of Sotagliflozin on Cardiovascular and Renal Events in Patients with Type 2 Diabetes and Moderate Renal Impairment Who Are at Cardiovascular Risk) [79]	Secondary endpoint	Primary endpoint
Empagliflozin	EMPA-KIDNEY (The Study of Heart and Kidney Protection with Empagliflozin) [80]	Secondary endpoint	Secondary endpoint
Dapagliflozin	DAPA-CKD (Dapagliflozin and Prevention of Adverse Outcomes in Chronic Kidney Disease) [78]	Secondary endpoint	Secondary endpoint

Some drugs are beneficial in reducing the risk of renal disease worsening as a secondary endpoint, but only CV benefits are shown. CV = cardiovascular, GLP-1 = glucagon-like peptide, HF = heart failure, SGLT2 = sodium-glucose cotransporter type 2, T2DM = type 2 diabetes mellitus. ** Clinical trial with semaglutide is ongoing (Clinicaltrials.gov number, NCT03819153) in patients with chronic kidney disease. † Major CV events included nonfatal myocardial infarction, nonfatal strokes, and death from CV disease. ‡ These drugs have a Food and Drug Administration label indication mentioning a reduction in this CV endpoint in the specific population of patients classified as patients with T2DM. ǁ Primary endpoint included HF hospitalization and CV death (and in DAPA-HF, HF emergency visit for HF).

## Data Availability

Not applicable.
